# Supplementary material for: A systematic review of pediatric clinical trials of high dose vitamin D
Source: PeerJ. 2016 Feb 25;4:e1701. doi: 10.7717/peerj.1701 (PMC4782742; doi:10.7717/peerj.1701)
Supplement: Table S2 [file peerj-04-1701-s005.doc]

| **Level** | **Screening Criteria** |
| --- | --- |
| **Level 1** | 1. The citation is not a review article (or case report) 2. The study is on humans and children are included 3. The study administers at least one dose of vitamin D (cholecalciferol, ergocalciferol) to the patient 4. The citation does not represent a conference abstract |
| **Level 2** | 1. At least one study arm (group) includes children 2. At least one dose of vitamin D (ergocalciferol and/or cholecalciferol) was administered 3. Vitamin D was administered at one or more doses determined by the investigators 4. The citation is in English, French, German or Spanish |
| **Level 3** | 1. One or more study arms provide vitamin D supplementation at a dose that r exceeds the IOM age specific Recommended Dietary Allowance (RDA) or Adequate Intake (AI) 2. If the study included adults, the information for pediatrics population is presented separately 3. Vitamin D was not administered mixed with food and in uncontrolled volume. |
