# Supplementary material for: A systematic review of pediatric clinical trials of high dose vitamin D
Source: PeerJ. 2016 Feb 25;4:e1701. doi: 10.7717/peerj.1701 (PMC4782742; doi:10.7717/peerj.1701)
Supplement: Table S3 [file peerj-04-1701-s006.doc]

| **Author** | **Year** | **Population** | **Supplementation** | **Measured Outcomes ^a^** |
| --- | --- | --- | --- | --- |
|  |  |  |  |  |
| **^b^ Arpadi, SM** | 2009 | HIV | 100000 D3 oral q2months | 25OHD, blood calcium, urine calcium, anthropometric measures, immune/inflammatory marker |
| **Arpadi, SM** | 2012 |  |  | 25OHD, blood calcium, urine calcium, bone mass marker |
|  |  |  |  |  |
| **^b^ Dahifar, H** | 2007 | Healthy/subclinical VDD | 50000 D2/3 oral daily | 25OHD, blood calcium, phosphate, ALP, PTH |
| **Dahifar, H** | 2006 |  |  | 25OHD, blood calcium, phosphate, ALP, PTH |
|  |  |  |  |  |
| **^b^ Kumar, GT** | 2011 | Premature and/or low birth weight | 1400 D3 oral weekly | 25OHD, anthropometric measures, adverse effects, hospital admission, mortality |
| **Kumar, GT** | 2012 |  |  | 25OHD, immune/inflammatory marker |
|  |  |  |  |  |
| **^b^ Leger, J** | 1989 | Hypothyroidism | 1200 D2 oral daily | 25OHD, 125(OH)2D, blood calcium, phosphate, thyroxine |
| **Tau, C** | 1986 |  |  | 25OHD, 125(OH)2D, blood calcium, phosphate, clinical rickets, ALP, anthropometric measures, thyroxine |
|  |  |  |  |  |
| **^b^ Manaseki-Holland, S** | 2012 | Healthy/subclinical VDD | 100000 D3 oral q4months | 25OHD, anthropometric measures, respiratory marker, adverse effects, clinical pneumonia, hospital admission, mortality |
| **Aluisio, AR** | 2013 |  |  | 25OHD, respiratory marker, adverse effects, diarrhea |
|  |  |  |  |  |
| **^b^ Marchisio, P** | 2013 | Recurrent acute otitis media | 1000 D3 oral daily | 25OHD, immune/inflammatory marker, adverse effects, clinical acute otitis media |
| **Principi, N** | 2013 |  |  | 25OHD, immune/inflammatory marker, adverse effects |

Abbreviations: Alkaline phosphatase (ALP), cholecalciferol (D3), ergocalciferol (D2), parathyroid hormone (PTH), every two months (q2months), vitamin D deficiency (VDD)

^a^ For outcome assessment in the rest of this study, we combined all measured outcomes between the two published papers.

^b^ Chosen paper for this publication.
