# Supplementary material for: A systematic review of pediatric clinical trials of high dose vitamin D
Source: PeerJ. 2016 Feb 25;4:e1701. doi: 10.7717/peerj.1701 (PMC4782742; doi:10.7717/peerj.1701)
Supplement: Table S4 — 181 populations received high dose of vitamin D. These populations were classified as conditions classically or non-classically associated with vitamin D deficiency. Outcomes were also indicated when they are different from the clinical diagnosis. [file peerj-04-1701-s007.doc]

| **Diagnostic Category / Outcome** | **Populations** |
| --- | --- |
| **Healthy/subclinical VDD with bone health as outcome** | **62** |
| **Classical** |  |
| Rickets | 27 |
| Premature and/or low birth weight | 24 |
| Epilepsy/seizure | 6 |
| Renal disease | 4 |
| Malabsorption, chronic cholestasis | 4 |
| Malabsorption, cystic fibrosis | 3 |
| Malabsorption | 1 |
| Malabsorption, biliary atresia | 1 |
| Malabsorption, crohn's disease | 1 |
| Malabsorption, inflammatory bowel disease | 1 |
| Malnutrition | 1 |
| Cerebral palsy/neuromuscular disorder | 1 |
| Growth hormone deficiency | 1 |
| Juvenile arthritis | 1 |
| Osteogenesis imperfecta | 1 |
| **Non-classical** |  |
| Obesity | 7 |
| Asthma | 4 |
| Healthy/subclinical VDD / dental fluorosis | 3 |
| Healthy/subclinical VDD / diabetes | 2 |
| Healthy/subclinical VDD / hematological outcomes | 2 |
| Atopic dermatitis | 2 |
| Divers conditions | 2 |
| HIV | 2 |
| Pneumonia | 2 |
| Recurrent acute otitis media | 2 |
| Beta-thalassemia | 1 |
| Cerebral palsy/neuromuscular disorder | 1 |
| Congestive heart failure | 1 |
| Healthy/subclinical VDD / acute otitis media | 1 |
| Healthy/subclinical VDD / cardiovascular | 1 |
| Healthy/subclinical VDD / influenza | 1 |
| Healthy/subclinical VDD / pneumonia | 1 |
| Healthy/subclinical VDD / tuberculosis | 1 |
| Hypothyroidism | 1 |
| Renal disease / hematological outcomes | 1 |
| Renal disease / renal outcomes | 1 |
| Rickets / antioxidants levels | 1 |
| Sickle cell | 1 |
| Tuberculosis | 1 |

Abbreviations: Vitamin D deficiency (VDD).
