# Supplementary material for: A systematic review of pediatric clinical trials of high dose vitamin D
Source: PeerJ. 2016 Feb 25;4:e1701. doi: 10.7717/peerj.1701 (PMC4782742; doi:10.7717/peerj.1701)
Supplement: Table S5 [file peerj-04-1701-s008.docx]

| Review | Abstracts | Full text | Ineligible | Online DB | Reduction (%) |
| --- | --- | --- | --- | --- | --- |
| Das, RR 2013 | 1343 | 32 | 30 | 2 | 30 (93.8%) |
| Fares, MM 2015 | 983 | 274 | 270 | 2 | 272 (99.3%) |
| Riverin, BD 2015 | 684 | 21 | 14 | 5 | 16 (76.2%) |
| Ali, SR 2015 | 818 ^a^ | 35 | 32 | 10 | 25 (71.4%) |

^a^ Number wasn’t provided in SR. Identified using their search strategy in Medline + Embase, and limiting the results to citations prior to their search date.
