# Supplementary material for: A systematic review of pediatric clinical trials of high dose vitamin D
Source: PeerJ. 2016 Feb 25;4:e1701. doi: 10.7717/peerj.1701 (PMC4782742; doi:10.7717/peerj.1701)
Supplement: Table S6 [file peerj-04-1701-s009.docx]

| Accessibility Measure | Mean ± SEM |
| --- | --- |
| **Page Setup** | **Total : 6/15 , 40 %** |
| HTTP-Equiv Content-Type (in header) | 0 |
| HTML Language Definition | 0 |
| Page Title | 3 |
| Meta Tag Keywords | 0 |
| Document type definition | 3 |
|  |  |
| **Access Restriction** | **Total : 12/12 , 100 %** |
| Image Alt Tags | 3 |
| Specified Image Widths | 3 |
| Table Summaries | 3 |
| Frames | 3 |
|  |  |
| **Outdated Code** | **Total : 27/27 , 100 %** |
| Body Tags - Body Background Colour | 3 |
| Body Tags - Body Topmargin | 3 |
| Body Tags - Body Margin Height | 3 |
| Table Tags - Table Background Colour | 3 |
| Table Tags - Table Column (td) Height | 3 |
| Table Tags - Table Row (tr) Height | 3 |
| Font Tags - Font Color | 3 |
| Font Tags - Font Size | 3 |
| Align (non style sheet) | 3 |
| Total | 45/54, 83.3% |
