# Supplementary material for: A systematic review of pediatric clinical trials of high dose vitamin D
Source: PeerJ. 2016 Feb 25;4:e1701. doi: 10.7717/peerj.1701 (PMC4782742; doi:10.7717/peerj.1701)
Supplement: Table S7 [file peerj-04-1701-s010.docx]

| Article | Year | Location | Patients | Population details | Risk of bias | Age | Regimen | All outcomes |
| --- | --- | --- | --- | --- | --- | --- | --- | --- |
| Wagner, VE. Effect of oral and intramuscular massive vitamin D doses on serum calcium in premature infants. [German].. Wiener Medizinische Wochenschrift | 1969 | Europe | 37 | Premature and/or low birth weight | Medium/Unclear | 21-28 days | 100000 D3 oral once | Blood calcium |
|  | 1969 | Europe | 31 | Premature and/or low birth weight | Medium/Unclear | 21-28 days | 100000 D3 IM once | Blood calcium |
| Gebre-Medhin, M. Effect of a single massive dose vitamin D therapy (oral or intramuscular) on rickets in Addis Ababa children.. Courrier | 1972 | Africa | 114 | Rickets | Medium/Unclear | 0-3 years | 250000 D2 IM once | Clinical Rickets, ALKP |
|  | 1972 | Africa | 113 | Rickets | Medium/Unclear | 0-3 years | 250000 D2 oral once | Clinical Rickets, ALKP |
|  | 1972 | Africa | 70 | Rickets | Medium/Unclear | 0-3 years | 10000 D3 oral Daily | Clinical Rickets, ALKP |
| Silver, J. Prevalence and treatment of vitamin D defeciency in children on anticonvulsant drugs. Archives of Disease in Childhood | 1974 | Europe | 33 | Epilepsy/Seizure | High | 10-16 years | 3000 D3 oral Weekly | Blood calcium, Phosphate, Clinical Rickets, Bone mass marker |
| Christiansen, C. Biochemical status in epileptic patients during treatment with vitamin D. A controlled therapeutic trial. Acta Neurologica Scandinavica | 1975 | Europe | 8 | Epilepsy/Seizure | Medium/Unclear | 7-20 years | 4000 D2 oral Daily | Blood calcium, ALKP |
| Radde, IC. Growth and mineral metabolism in very low birth weight infants. I. Comparison of the effects of two modes of NaHCO3 treatment of late metabolic acidosis. Pediatric Research | 1975 | North America | 14 | Premature and/or low birth weight | Medium/Unclear | 14-14 days | 400 D2 oral Daily + NaHCO3 | Blood calcium, Bone mass marker, other - (see detailed form) |
|  | 1975 | North America | 12 | Premature and/or low birth weight | Medium/Unclear | 14-14 days | 400 D2 oral Daily + Lower dose of NaHCO3 | Blood calcium, Bone mass marker, other - (see detailed form) |
| Day, GM. Growth and mineral metabolism in very low birth weight infants. II. Effects of calcium supplementation on growth and divalent cations.. Pediatric Research | 1975 | North America | 16 | Premature and/or low birth weight | High | 14-21 days | 500 D2 oral Daily | Blood calcium, Urine calcium, Phosphate, Clinical Rickets, other - (see detailed form) |
|  | 1975 | North America | 14 | Premature and/or low birth weight | High | 14-21 days | 500 D2 oral Daily + Calcium | Blood calcium, Urine calcium, Phosphate, Clinical Rickets, other - (see detailed form) |
| Christiansen, C. Iatrogenic osteomalacia in epileptic children. A controlled therapeutic trial. Acta Paediatrica Scandinavica | 1975 | Europe | 14 | Epilepsy/Seizure | Medium/Unclear | 7-14 years | 2000 D2 oral Daily | Blood calcium, Bone mass marker, ALKP |
| Liakakos, D. Serum alkaline phosphatase and urinary hydroxyproline values in children receiving phenobarbital with and without vitamin D.. Journal of Pediatrics | 1975 | Europe | 8 | Epilepsy/Seizure | High | 5-14 years | 200000 D2 oral once | Clinical Rickets, Bone mass marker, ALKP, other - (see detailed form) |
|  | 1975 | Europe | 8 | Epilepsy/Seizure | High | 5-14 years | 4000 D2 oral Daily | Clinical Rickets, Bone mass marker, ALKP, other - (see detailed form) |
|  | 1975 | Europe | 8 | Epilepsy/Seizure | High | 5-14 years | 4000 D2 oral Daily | Clinical Rickets, Bone mass marker, ALKP, other - (see detailed form) |
| Moya, M. Therapeutic and collateral effects of 25-hydroxycholecalciferol in vitamin D deficiency. European Journal Pediatrics | 1977 | Europe | 9 | Rickets | Medium/Unclear | 3-18 months | 6000 D3 Daily | Blood calcium, Urine calcium, Phosphate, Clinical Rickets, ALKP, other - (see detailed form) |
| Holst-Gemeiner, D. Plasma 25-hydroxycholecalciferol after daily vitamin D administration in comparison with massive single-dose prophylaxis. Wiener Klinische Wochenschrift | 1978 | Europe | 10 | Healthy/Subclinical VDD | Medium/Unclear | 0-8 days | 1200 D3 oral Daily | 25OHD |
|  | 1978 | Europe | 11 | Healthy/Subclinical VDD | Medium/Unclear | 0-8 days | 200000 D3 oral once | 25OHD |
| Robinson, MJ. Plasma 25 hydroxyvitamin D concentrations in preterm infants receiving oral vitamin D supplements.. Archives of Disease in Childhood | 1981 | Europe | 9 | Premature and/or low birth weight | Medium/Unclear | 15-15 days | 400 D3 oral Daily | 25OHD, Blood calcium, Clinical Rickets, ALKP |
|  | 1981 | Europe | 9 | Premature and/or low birth weight | Medium/Unclear | 15-15 days | 1000 D3 oral Daily | 25OHD, Blood calcium, Clinical Rickets, ALKP |
| Glorieux, FH. Vitamin D metabolism in preterm infants: serum calcitriol values during the first five days of life.. Journal of Pediatrics | 1981 | Europe | 8 | Premature and/or low birth weight | Medium/Unclear | 0-0 days | 2100 D3 oral Daily | 25OHD , 125(OH)2D, Blood calcium, Phosphate, PTH |
| Kunz, C. 25-hydroxy-vitamin-D in serum of newborns and infants during continuous oral vitamin D treatment. Padiatrie und Padologie | 1982 | Europe | 13 | Healthy/Subclinical VDD | Medium/Unclear | 0-1 months | 500 D2/3 oral Daily | 25OHD, Blood calcium, PTH |
|  | 1982 | Europe | 16 | Healthy/Subclinical VDD | Medium/Unclear | 0-1 months | 1000 D2/3 oral Daily | 25OHD, Blood calcium, PTH |
| Raghuramulu, N. Studies on vitamin D metabolism in malnourished children. British Journal of Nutrition | 1982 | East Asia | 5 | Healthy/Subclinical VDD | Medium/Unclear | 1-5 years | 600000 D3 oral once | 25OHD |
| Raghuramulu N. Studies on vitamin D metabolism in malnourished children. British Journal of Nutrition | 1982 | East Asia | 14 | Malnutrition | Medium/Unclear | 1-5 years | 600000 D3 oral once | 25OHD |
| Raghuramulu, N. Studies on vitamin D metabolism in malnourished children. British Journal of Nutrition | 1982 | East Asia | 5 | Healthy/Subclinical VDD | Medium/Unclear | 1-5 years | 2000 D3 oral Daily | 25OHD |
| Raghuramulu N. Studies on vitamin D metabolism in malnourished children. British Journal of Nutrition | 1982 | East Asia | 13 | Malnutrition | Medium/Unclear | 1-5 years | 2000 D3 oral Daily | 25OHD |
| Eke, FU. Effect of low dose 1 alpha-hydroxycholecalciferol on glomerular filtration rate in moderate renal failure. Archives of Disease in Childhood | 1983 | Europe | 8 | Renal disease | Medium/Unclear | 6.5-18 year | 26 IU/kg D3 oral Daily | Blood calcium, Phosphate, Bone mass marker, PTH, Renal marker, other - (see detailed form) |
| Westfechtel. Hypophosphatemic rickets in premature infants weighing less than 1500 grams on oral and parenteral feeding. [German].. Monatsschrift Kinderheilkunde | 1984 | Europe | 9 | Premature and/or low birth weight | High | 0-0 days | 400 D3 IV Daily | 25OHD, Phosphate, ALKP |
|  | 1984 | Europe | 16 | Premature and/or low birth weight | High | 0-0 days | 1000 D3 oral Daily | 25OHD, Phosphate, ALKP |
| Markestad, T. Plasma concentrations of vitamin D metabolites before and during treatment of vitamin D deficiency rickets in children. Acta Paediatrica Scandinavica | 1984 | Europe | 17 | Rickets | High | not stated | 1700-4000 D2 oral Daily | 25OHD , 125(OH)2D, Blood calcium, Phosphate, ALKP, PTH, other - (see detailed form) |
| Ala-Houhala, M. 25-Hydroxyvitamin D levels during breast-feeding with or without maternal or infantile supplementation of vitamin D. Journal of Pediatric Gastroenterology & Nutrition | 1985 | Europe | 29 | Healthy/Subclinical VDD | Medium/Unclear | 0-7 days | 1000 D2/3 oral Daily | 25OHD, Blood calcium, Phosphate, ALKP, other - (see detailed form) |
| Hillman, LS. Absorption, dosage, and effect on mineral homeostasis of 25-hydroxycholecalciferol in premature infants: comparison with 400 and 800 IU vitamin D2 supplementation.. Journal of Pediatrics | 1985 | North America | 21 | Premature and/or low birth weight | High | 0-2 months | 400 D2 oral Daily | 25OHD , 125(OH)2D, Blood calcium, Urine calcium, Phosphate, Bone mass marker, PTH |
|  | 1985 | North America | 15 | Premature and/or low birth weight | High | 0-2 months | 800 D2 oral Daily | 25OHD , 125(OH)2D, Blood calcium, Urine calcium, Phosphate, Bone mass marker, PTH |
| Hodson, EM. Treatment of childhood renal osteodystrophy with calcitriol or ergocalciferol.. Clinical Nephrology | 1985 | Australia/New Zealand | 11 | Renal disease | High | not stated | 6000 - 110000 D2 oral Daily | 25OHD, Blood calcium, Phosphate, Clinical Rickets, Bone mass marker, ALKP, PTH, other - (see detailed form) |
| Stogmann, W. Vitamin D deficiency rickets: single-dose therapy versus continuous therapy. Padiatrie und Padologie | 1985 | Europe | 5 | Rickets | Medium/Unclear | not stated | 200000 IU D2 oral Intermittent | 25OHD, Blood calcium, Phosphate, ALKP, PTH, other - (see detailed form) |
|  | 1985 | Europe | 5 | Rickets | Medium/Unclear | not stated | 9600 D2 oral Daily | 25OHD, Blood calcium, Phosphate, ALKP, PTH, other - (see detailed form) |
| Goncerzewicz, M. Vitamin D metabolism in children with malabsorption syndrome. Klinische Padiatrie | 1985 | Europe | 15 | Malabsorption | Medium/Unclear | 0-18 years | 1200 IU/kg D3 oral once | 25OHD, other - (see detailed form) |
|  | 1985 | Europe | 15 | Malabsorption | Medium/Unclear | 0-18 years | 12000 IU/kg D3 oral once | 25OHD, other - (see detailed form) |
| Tau, C. Hypercalcemia in infants with congenital hypothyroidism and its relation to vitamin D and thyroid hormones. The Journal of Pediatrics | 1986 | Europe | 12 | Other, Hypothyroidism | Medium/Unclear | 0-1 months | 1200 D2 oral Daily | 25OHD , 125(OH)2D, Blood calcium, Phosphate, Clinical Rickets, ALKP, Anthropometric measures, other - (see detailed form) |
| Pettifor, JM. Mineral homeostasis in very low birth weight infants fed either own mother's milk or pooled pasteurized preterm milk.. Journal of Pediatric Gastroenterology & Nutrition | 1986 | Africa | 33 | Premature and/or low birth weight | Medium/Unclear | 4-4 days | 750 D2 oral Daily (Own-mother’s milk) | 25OHD, Blood calcium, Phosphate, Bone mass marker, ALKP, other - (see detailed form) |
|  | 1986 | Africa | 35 | Premature and/or low birth weight | Medium/Unclear | 4-4 days | 750 D2 oral Daily (Pooled milk) | 25OHD, Blood calcium, Phosphate, Bone mass marker, ALKP, other - (see detailed form) |
| Mawer, EB. Vitamin D nutrition and vitamin D metabolism in the premature human neonate.. Clinical Endocrinology | 1986 | Europe | 19 | Premature and/or low birth weight | High | 0-21 days | 1000 D2 oral Daily | 25OHD , 125(OH)2D, Blood calcium |
|  | 1986 | Europe | 19 | Premature and/or low birth weight | High | 0-21 days | 3000 D2 oral Daily | 25OHD , 125(OH)2D, Blood calcium |
| Markestad, T. Intermittent high-dose vitamin D prophylaxis during infancy: effect on vitamin D metabolites, calcium, and phosphorus. American Journal of Clinical Nutrition | 1987 | Europe | 43 | Healthy/Subclinical VDD | High | 1-20 months | 600000 D2 oral once | 25OHD , 125(OH)2D, Blood calcium, Phosphate, other - (see detailed form) |
| Schmidt, P. Preliminary experiences in the prevention of rickets with reduced vitamin D doses. Kinderarztliche Praxis | 1988 | Europe | 40 | Healthy/Subclinical VDD | High | 0-1 months | D3 IM Intermittent: 200000 IU at 10-14 days of life, followed by 9000 IU weekly until 6 months and then 6000 IU weekly for the remaining 6 months | Blood calcium, Urine calcium, Phosphate |
|  | 1988 | Europe | 40 | Healthy/Subclinical VDD | High | 0-1 months | 6000 D3 oral Weekly | Blood calcium, Urine calcium, Phosphate |
|  | 1988 | Europe | 40 | Healthy/Subclinical VDD | High | 0-1 months | 3000 D3 oral Weekly | Blood calcium, Urine calcium, Phosphate |
| Heubi, JE. Bone disease in chronic childhood cholestasis. I. Vitamin D absorption and metabolism.. Hepatology | 1989 | North America | 6 | Malabsorption, Chronic cholestasis | High | not stated | 1000 IU/Kg D2 oral once | 25OHD , 125(OH)2D, Blood calcium, Phosphate, Clinical Rickets, Bone mass marker, PTH, other - (see detailed form) |
|  | 1989 | North America | 6 | Other, Random Conditions | High | not stated | 1000 IU/kg D2 oral once | 25OHD , 125(OH)2D, Blood calcium, Phosphate, Clinical Rickets, Bone mass marker, PTH, other - (see detailed form) |
| Pettifor, JM. Bone mineralization and mineral homeostasis in very low-birth-weight infants fed either human milk or fortified human milk.. Journal of Pediatric Gastroenterology and Nutrition | 1989 | Africa |  | Premature and/or low birth weight | Medium/Unclear | 4-4 days | 750 D2 oral Daily (Breast-fed) | 25OHD , 125(OH)2D, Blood calcium, Urine calcium, Phosphate, Bone mass marker, ALKP |
|  | 1989 | Africa |  | Premature and/or low birth weight | Medium/Unclear | 4-4 days | 750 D2 oral Daily (Fortified with formula) | 25OHD , 125(OH)2D, Blood calcium, Urine calcium, Phosphate, Bone mass marker, ALKP |
| Evans, JR. Effect of high-dose vitamin D supplementation on radiographically detectable bone disease of very low birth weight infants.. Journal of Pediatrics | 1989 | North America | 42 | Premature and/or low birth weight | Low | 3-3 days | 400 D2 oral Daily | 25OHD, Blood calcium, Urine calcium, Phosphate, Clinical Rickets, ALKP |
|  | 1989 | North America | 45 | Premature and/or low birth weight | Low | 3-3 days | 2000 D2 oral Daily | 25OHD, Blood calcium, Urine calcium, Phosphate, Clinical Rickets, ALKP |
| Masood, H. Persistent limb pain and raised serum alkaline phosphatase the earliest markers of subclinical hypovitaminosis D in Kashmir. Indian Journal of Physiology & Pharmacology | 1989 | East Asia | 100 | Healthy/Subclinical VDD | High | 5-14 years | 300000 D2/3 IM once | Blood calcium, Phosphate, ALKP, Renal marker, Hematology, Immune/Inflammatory marker, other - (see detailed form) |
| Leger, J. Prophylaxis of vitamin D deficiency in hypothyroidism in the newborn infant. Archives Francaises de Pediatrie | 1989 | Europe | 11 | Other, Hypothyroidism | Medium/Unclear | 0-1 months | 1200 D2 oral Daily | 25OHD , 125(OH)2D, Blood calcium, Phosphate, other - (see detailed form) |
| Reinken, L. The effect of osteopenia prevention in very small premature infants on hormonal parameters of calcium metabolism and bone mineralization[German].. Klinische Padiatrie | 1989 | Europe | 8 | Premature and/or low birth weight | Medium/Unclear | 0-0 days | 1000 D3 oral Daily (before 28 days of age) | 25OHD, Blood calcium, Phosphate, ALKP, PTH, other - (see detailed form) |
|  | 1989 | Europe | 7 | Premature and/or low birth weight | Medium/Unclear | 0-0 days | 1000 D3 oral Daily (after 28 days of age) | 25OHD, Blood calcium, Phosphate, ALKP, PTH, other - (see detailed form) |
|  | 1989 | Europe | 8 | Premature and/or low birth weight | Medium/Unclear | 0-0 days | 1000 D3 oral Daily | 25OHD, Blood calcium, Phosphate, ALKP, PTH, other - (see detailed form) |
| Lubani, MM. Vitamin-D-deficiency rickets in Kuwait: the prevalence of a preventable disease. Annals of Tropical Paediatrics | 1989 | Middle East | 125 | Rickets | High | 1-24 months | 600000 D2/3 IM once | Blood calcium, Phosphate, Clinical Rickets, ALKP |
|  | 1989 | Middle East | 125 | Rickets | High | 1-24 months | D2/3 oral Daily: 2000 IU for 4 weeks, followed by 400IU/day until 2y of age or (in older children) for at least 1y following start of treatment | Blood calcium, Phosphate, Clinical Rickets, ALKP |
| Heubi, JE. Bone disease in chronic childhood cholestasis. II. Better absorption of 25-OH vitamin D than vitamin D in extrahepatic biliary atresia.. Pediatric research | 1990 | North America | 6 | Malabsorption, Chronic cholestasis | High | 0.33-1.83 y | 1000 IU/Kg D2 oral once | 25OHD , 125(OH)2D, Blood calcium, Phosphate, Clinical Rickets, Bone mass marker, PTH, other - (see detailed form) |
|  | 1990 | North America | 5 | Malabsorption, Chronic cholestasis | High | 1-8.33 year | 1000 IU/kg D2 oral once | 25OHD , 125(OH)2D, Blood calcium, Phosphate, Clinical Rickets, Bone mass marker, PTH, other - (see detailed form) |
|  | 1990 | North America | 6 | Other, Divers conditions | High | 10.1-19.1 y | 1000 IU/Kg D2 oral once | 25OHD , 125(OH)2D, Blood calcium, Phosphate, Clinical Rickets, Bone mass marker, PTH, other - (see detailed form) |
| Pittard III, WB. How much vitamin D for neonates?.. American journal of diseases of children | 1991 | North America | 8 | Premature and/or low birth weight | Medium/Unclear | 0-0 days | 400 D2/3 oral Daily | 25OHD, Blood calcium, Phosphate, PTH |
|  | 1991 | North America | 5 | Healthy/Subclinical VDD | Medium/Unclear | 0-0 days | 800 D2/3 oral Daily | 25OHD, Blood calcium, Phosphate, PTH |
|  | 1991 | North America | 9 | Premature and/or low birth weight | Medium/Unclear | 0-0 days | 800 D2/3 oral Daily | 25OHD, Blood calcium, Phosphate, PTH |
| Argao, EA. d-Alpha-tocopheryl polyethylene glycol-1000 succinate enhances the absorption of vitamin D in chronic liver disease of infancy and childhood. Pediatric Research | 1992 | North America | 8 | MalabsorptionChronic childhood cholestas | High | 0.4-19 year | 1000 IU/Kg D3 oral Daily | 25OHD, Blood calcium, Phosphate, Bone mass marker, other - (see detailed form) |
| Gupta, SK. Reversal of clinical and dental fluorosis.. Indian Pediatrics | 1994 | East Asia | 20 | Healthy/Subclinical VDD | High | 3-12 years | 800 D3 oral Daily. (Vanasthali: 8.5 ppm Fluorides in water) | Blood calcium, ALKP, other - (see detailed form) |
|  | 1994 | East Asia | 20 | Healthy/Subclinical VDD | High | 3-12 years | 800 D3 oral Daily. (Shivdaspura: 4.5 ppm Fluorides in water) | Blood calcium, ALKP, other - (see detailed form) |
| Zuckerman, M. Rickets in very-low-birth-weight infants born at Baragwanath Hospital.. South African Medical Journal | 1994 | Africa | 27 | Premature and/or low birth weight | Medium/Unclear | 14-14 days | 800 D2/3 oral Daily (Breast feed) | 25OHD , 125(OH)2D, Blood calcium, Phosphate, Clinical Rickets, ALKP, PTH, other - (see detailed form) |
|  | 1994 | Africa | 29 | Premature and/or low birth weight | Medium/Unclear | 14-14 days | 800 D2/3 oral Daily (Formula) | 25OHD , 125(OH)2D, Blood calcium, Phosphate, Clinical Rickets, ALKP, PTH, other - (see detailed form) |
| Zeghoud, F. Vitamin D prophylaxis during infancy: comparison of the long-term effects of three intermittent doses (15,5, or 2.5 mg) on 25-hydroxyvitamin D concentrations. American Journal of Clinical Nutrition | 1994 | Africa | 30 | Healthy/Subclinical VDD | High | 15-15 days | 600000 D3 oral once | 25OHD, Blood calcium, Phosphate |
|  | 1994 | Africa | 15 | Healthy/Subclinical VDD | Medium/Unclear | 1-1 days | 100000 D3 oral q3months | 25OHD, Blood calcium, Phosphate |
|  | 1994 | Africa | 15 | Healthy/Subclinical VDD | Medium/Unclear | 1-1 days | 200000 D3 oral once | 25OHD, Blood calcium, Phosphate |
| Zeghoud, F. Vitamin D and pubertal maturation. Value and tolerance of vitamin D supplementation during the winter season. Archives de pÃ©diatrie | 1995 | Europe | 9 | Healthy/Subclinical VDD | High | 10-17 years | 100000 D3 oral once | 25OHD , 125(OH)2D, Blood calcium, PTH |
| Edda, LF. Effects of two forms of vitamin D supplementation on bone metabolism and infants growth. Revista Chilena de Pediatria | 1996 | Central & South America | 35 | Healthy/Subclinical VDD | Medium/Unclear | 15-30 days | 600000 D3 oral q5months | 125(OH)2D, Blood calcium, Phosphate, PTH, Anthropometric measures, Cardiovascular marker, other - (see detailed form) |
| Gupta, SK. Reversal of fluorosis in children.. Acta Paediatrica Japonica | 1996 | East Asia | 15 | Healthy/Subclinical VDD | Medium/Unclear | 6-12 years | 60000 D3 oral Weekly | Blood calcium, ALKP, other - (see detailed form) |
| Oliveri, B. Vitamin D prophylaxis in children with a single dose of 150000 IU of vitamin D. European Journal of Clinical Nutrition | 1996 | Central & South America | 79 | Healthy/Subclinical VDD | High | 5-11 years | 150000 D2 oral once | 25OHD, Blood calcium, Urine calcium, Phosphate, ALKP, PTH |
| Vervel, C. Fortified milk and supplements of oral vitamin D. Comparison of the effect of two doses of vitamin D (500 and 1,000 UI/d) during the first trimester of life. Archives de Pediatrie | 1997 | Europe | 40 | Healthy/Subclinical VDD | Medium/Unclear | 3-6 days | 500 D2 oral Daily | 25OHD, PTH |
|  | 1997 | Europe | 40 | Healthy/Subclinical VDD | Medium/Unclear | 3-6 days | 1000 D2 oral Daily | 25OHD, PTH |
| Hoppe, B. Influence of nutrition on urinary oxalate and calcium in preterm and term infants. Pediatric Nephrology | 1997 | Europe | 37 | Healthy/Subclinical VDD | High | 0-2 months | 500 D3 oral Daily | Blood calcium, Urine calcium, Bone mass marker, other - (see detailed form) |
| Zeghoud, F. Subclinical vitamin D deficiency in neonates: definition and response to vitamin D supplements. American Journal of Clinical Nutrition | 1997 | Europe | 40 | Healthy/Subclinical VDD | Medium/Unclear | 0-6 days | 500 D2 oral Daily | 25OHD, Blood calcium, Phosphate, ALKP, PTH |
|  | 1997 | Europe | 40 | Healthy/Subclinical VDD | Medium/Unclear | 0-6 days | 1000 D2 oral Daily | 25OHD, Blood calcium, Phosphate, ALKP, PTH |
| Morcos, MM. Vitamin D administration to tuberculous children and its value. Bollettino Chimico Farmaceutico | 1998 | Middle East | 12 | Other, Tuberculosis | Medium/Unclear | not stated | 1000 D2/3 oral Daily | 125(OH)2D, Blood calcium |
| Guillemant, J. Vitamin D status in the adolescent: seasonal variations and effects of winter supplementation with vitamin D3. Archives de Pediatrie | 1998 | Europe | 24 | Healthy/Subclinical VDD | Medium/Unclear | 13-16 years | 100000 D3 oral q2months | 25OHD, PTH |
| Thacher, TD. A comparison of calcium, vitamin D, or both for nutritional rickets in Nigerian children. The New England Journal of Medicine | 1999 | Africa | 41 | Rickets | Low | 1-14 years | 600000 D2/3 IM q3months | 25OHD, Blood calcium, Clinical Rickets, ALKP |
|  | 1999 | Africa | 41 | Rickets | Low | 1-14 years | 600000 D2/3 IM q3months + Calcium | 25OHD, Blood calcium, Clinical Rickets, ALKP |
| Backstrom, MC. Randomised controlled trial of vitamin D supplementation on bone density and biochemical indices in preterm infants.. Archives of Disease in Childhood Fetal &amp; Neonat | 1999 | Europe | 21 | Premature and/or low birth weight | Medium/Unclear | 0-2 days | 200 IU/Kg D2/3 oral Daily | 25OHD , 125(OH)2D, Blood calcium, Phosphate, Bone mass marker, ALKP, PTH, Anthropometric measures |
|  | 1999 | Europe | 18 | Premature and/or low birth weight | Medium/Unclear | 0-2 days | 960 D2/3 oral Daily | 25OHD , 125(OH)2D, Blood calcium, Phosphate, Bone mass marker, ALKP, PTH, Anthropometric measures |
| Backstrom, MC. The long-term effect of early mineral, vitamin D, and breast milk intake on bone mineral status in 9- to 11-year-old children born prematurely.. Journal of Pediatric Gastroenterology and Nutrition | 1999 | Europe | 22 | Premature and/or low birth weight | Medium/Unclear | 0-0 days | 500 D2/3 oral Daily | 25OHD , 125(OH)2D, Bone mass marker, Anthropometric measures |
|  | 1999 | Europe | 12 | Premature and/or low birth weight | Medium/Unclear | 0-0 days | 500 D2/3 oral Daily + Phosphate | 25OHD , 125(OH)2D, Bone mass marker, Anthropometric measures |
|  | 1999 | Europe | 23 | Premature and/or low birth weight | Medium/Unclear | 0-0 days | 1000 D2/3 oral Daily | 25OHD , 125(OH)2D, Bone mass marker, Anthropometric measures |
|  | 1999 | Europe | 13 | Premature and/or low birth weight | Medium/Unclear | 0-0 days | 1000 D2/3 oral Daily + Phosphate | 25OHD , 125(OH)2D, Bone mass marker, Anthropometric measures |
| Baroncelli, GI. Bone turnover in children with vitamin D deficiency rickets before and during treatment. Acta Paediatrica | 2000 | Europe | 14 | Rickets | High | not stated | 3000-4000 D3 oral Daily | 25OHD, Blood calcium, Phosphate, Bone mass marker, ALKP, other - (see detailed form) |
| Duhamel, JF. Prevention of vitamin D deficiency in adolescents and pre-adolescents. An interventional multicenter study on the biological effect of repeated doses of 100,000 IU of vitamin D3. Archives de Pediatrie | 2000 | Europe | 33 | Healthy/Subclinical VDD | Low | 10-15 years | 100000 D3 oral q3months | 25OHD, Blood calcium, Urine calcium, Phosphate, Bone mass marker, ALKP, PTH |
| Tiosano, D. The role of the vitamin D receptor in regulating vitamin D metabolism: a study of vitamin D-dependent rickets, type II.. Journal of Clinical Endocrinology & Metabolism | 2001 | Middle East | 7 | Healthy/Subclinical VDD | High | 4-16 years | 50000 IU/m2 D3 oral once | 25OHD , 125(OH)2D, Blood calcium, Urine calcium, Phosphate, ALKP, PTH, Renal marker, other - (see detailed form) |
|  | 2001 | Middle East | 7 | Rickets | High | 4-16 years | 50000 IU/m2 D3 oral once (PTH normal, Urine calcium high) | 25OHD , 125(OH)2D, Blood calcium, Urine calcium, Phosphate, ALKP, PTH, Renal marker, other - (see detailed form) |
|  | 2001 | Middle East | 3 | Rickets | High | 4-16 years | 50000 IU/m2 D3 oral once (PTH high, Urine calcium normal) | 25OHD , 125(OH)2D, Blood calcium, Urine calcium, Phosphate, ALKP, PTH, Renal marker, other - (see detailed form) |
| Guillemant, J. Wintertime vitamin D deficiency in male adolescents: effect on parathyroid function and response to vitamin D3 supplements. Osteoporosis International | 2001 | Europe | 29 | Healthy/Subclinical VDD | Medium/Unclear | 13-17 years | 100000 D3 oral q2months | 25OHD, PTH |
| Kutluk, G. Comparisons of oral calcium, high dose vitamin D and a combination of these in the treatment of nutritional rickets in children. Journal of tropical pediatrics | 2002 | Middle East | 14 | Rickets | Medium/Unclear | not stated | 300000 D2/3 IM once | Blood calcium, Phosphate, Clinical Rickets, Bone mass marker, ALKP |
|  | 2002 | Middle East | 14 | Rickets | Medium/Unclear | not stated | 300000 D2/3 IM once + Calcium | Blood calcium, Phosphate, Clinical Rickets, Bone mass marker, ALKP |
| Lehtonen-Veromaa, M. The effect of conventional vitamin D2 supplementation on serum 25(OH)D concentration is weak among peripubertal Finnish girls: A 3-y prospective study.. European Journal of Clinical Nutrition | 2002 | Europe | 191 | Healthy/Subclinical VDD | High | 11-17 years | 800 D2 oral Daily | 25OHD, Blood calcium, Phosphate, Bone mass marker, ALKP, other - (see detailed form) |
| Cesur, Y. Comparison of low and high dose of vitamin D treatment in nutritional vitamin D deficiency rickets. Journal of Pediatric Endocrinology & Metabolism | 2003 | Middle East | 20 | Rickets | Medium/Unclear | 3-36 months | 150000 D2/3 oral once | Blood calcium, Urine calcium, Phosphate, Clinical Rickets, ALKP |
|  | 2003 | Middle East | 20 | Rickets | Medium/Unclear | 3-36 months | 300000 D2/3 oral once | Blood calcium, Urine calcium, Phosphate, Clinical Rickets, ALKP |
|  | 2003 | Middle East | 16 | Rickets | Medium/Unclear | 3-36 months | 600000 D2/3 oral once | Blood calcium, Urine calcium, Phosphate, Clinical Rickets, ALKP |
| Pehlivan, L. Maternal vitamin D defiency and vitamin D supplementation in healthy infants.. Turkish Journal of Pediatrics | 2003 | Middle East | 21 | Healthy/Subclinical VDD | High | 7-7 days | 800 D2/3 oral Daily | 25OHD, Blood calcium, Phosphate, Clinical Rickets, ALKP |
| Balasubramanian K. Varying role of vitamin D deficiency in the etiology of rickets in young children vs. adolescents in northern India.. Journal of Tropical Pediatrics | 2003 | East Asia | 16 | Rickets | High | 120-240 mon | 600000 D2/3 oral once | 25OHD , 125(OH)2D, Blood calcium, Phosphate, ALKP |
| Balasubramanian, K. Varying role of vitamin D deficiency in the etiology of rickets in young children vs. adolescents in northern India.. Journal of Tropical Pediatrics | 2003 | East Asia |  | Rickets | High | 0-120 month | 6000 D2/3 oral Daily | 25OHD , 125(OH)2D, Blood calcium, Phosphate, ALKP |
|  | 2003 | East Asia |  | Rickets | High | 0-120 month | 600000 D2/3 oral once | 25OHD , 125(OH)2D, Blood calcium, Phosphate, ALKP |
| Hawker, GA. Alendronate in the treatment of low bone mass in steroid-treated boys with Duchennes muscular dystrophy.. Archives of Physical Medicine & Rehabilitation | 2005 | North America | 16 | Other, Cerebral palsy/Neuromuscular disorder | High | not stated | 1000 D2/3 oral Daily | Bone mass marker, Adverse effects |
| Forin, V. Benefits of pamidronate in children with osteogenesis imperfecta: An open prospective study.. Joint Bone Spine | 2005 | Europe | 29 | Other, Osteogenesis imperfecta | High | not stated | 1200 D2/3 oral Daily | 25OHD, Blood calcium, Urine calcium, Phosphate, Bone mass marker, ALKP, PTH, other - (see detailed form) |
| Delvin, EE. Oral vitamin A, E and D supplementation of pre-term newborns either breast-fed or formula-fed: a 3-month longitudinal study.. Journal of Pediatric Gastroenterology and Nutrition | 2005 | Europe | 23 | Premature and/or low birth weight | Medium/Unclear | not stated | 1000 D2/3 oral Daily (+ Breast feed) | 25OHD, other - (see detailed form) |
|  | 2005 | Europe | 62 | Premature and/or low birth weight | Medium/Unclear | not stated | 1000 D2/3 oral Daily (+Prenatal Formula) | 25OHD, other - (see detailed form) |
| Alizade, P. A randomized clinical trial of prophylactic effects of vitamin D on different indices of osteopenia of prematurity.. Iranian Journal of Public Health | 2006 | Middle East | 32 | Premature and/or low birth weight | Medium/Unclear | 0-14 days | 400 D2/3 oral Daily | Blood calcium, Phosphate, Clinical Rickets, Bone mass marker, ALKP |
|  | 2006 | Middle East | 36 | Premature and/or low birth weight | Medium/Unclear | 0-14 days | 1000 D2/3 oral Daily | Blood calcium, Phosphate, Clinical Rickets, Bone mass marker, ALKP |
| Akcam, M. Bone mineral density in response to two different regimes in rickets. Indian Pediatrics | 2006 | Middle East | 10 | Rickets | High | 5-13 months | 600000 D3 oral once | 25OHD, Blood calcium, Phosphate, Clinical Rickets, Bone mass marker, ALKP |
|  | 2006 | Middle East | 10 | Rickets | High | 5-13 months | 20000 D3 oral Daily | 25OHD, Blood calcium, Phosphate, Clinical Rickets, Bone mass marker, ALKP |
| Thacher, TD. Early response to vitamin D2 in children with calcium deficiency rickets. The Journal of Pediatrics | 2006 | Africa | 16 | Rickets | High | not stated | 50000 D2 oral once | 25OHD , 125(OH)2D, Blood calcium, Phosphate, ALKP, PTH |
| El-Hajj, FG. Effect of vitamin D replacement on musculoskeletal parameters in school children: A randomized controlled trial. The Journal of Clinical Endocrinology and Metabolis | 2006 | Middle East | 62 | Healthy/Subclinical VDD | Medium/Unclear | 10-17 years | 1400 D3 oral Weekly | 25OHD , 125(OH)2D, Blood calcium, Clinical Rickets, Bone mass marker, Anthropometric measures, other - (see detailed form) |
|  | 2006 | Middle East | 59 | Healthy/Subclinical VDD | Medium/Unclear | 10-17 years | 14000 D3 oral Weekly | 25OHD , 125(OH)2D, Blood calcium, Clinical Rickets, Bone mass marker, Anthropometric measures, other - (see detailed form) |
| Henriksen, C. Fat-soluble vitamins in breast-fed preterm and term infants.. European Journal of Clinical Nutrition | 2006 | Europe | 43 | Premature and/or low birth weight | High | 0-1 days | 500 D3 oral Daily | 25OHD |
| Wicklow, BA. Feasibility of a type 1 diabetes primary prevention trial using 2000 IU vitamin D3 in infants from the general population with increased HLA-associated risk.. Annals of the New York Academy of Sciences | 2006 | North America | 4 | Healthy/Subclinical VDD | High | 0-1 months | 2000 D3 oral Daily | 25OHD , 125(OH)2D, Blood calcium, Urine calcium, Phosphate, Bone mass marker, Diabetes marker, other - (see detailed form) |
| Dahifar, H. Impact of dietary and lifestyle on vitamin D in healthy student girls aged 11-15 years. The Journal of Medical Investigation | 2006 | Middle East | 7 | Healthy/Subclinical VDD | Medium/Unclear | 11-15 years | 50000 D2/3 oral Daily | 25OHD, Blood calcium, Phosphate, ALKP, PTH |
| Mikati, MA. Two randomized vitamin D trials in ambulatory patients on anticonvulsants: impact on bone. Neurology | 2006 | Middle East | 46 | Epilepsy/Seizure | Medium/Unclear | 10-18 years | 2000 D2 oral Daily | 25OHD, Bone mass marker |
| Dahifar, H. Asymptomatic rickets in adolescent girls. The Indian Journal of Pediatrics | 2007 | Middle East | 7 | Healthy/Subclinical VDD | Medium/Unclear | 11-15 years | 50000 D2/3 oral Daily | 25OHD, Blood calcium, Phosphate, ALKP, PTH |
| Kilpinen-Loisa, P. High-dose vitamin D supplementation in children with cerebral palsy or neuromuscular disorder. Neuropediatrics | 2007 | Europe | 22 | Other, Cerebral palsy/Neuromuscular disorder | High | 9-18 years | 1000 D3 oral Daily | 25OHD, Blood calcium, Phosphate, ALKP, PTH |
| Tau, C. Twice single doses of 100,000 IU of vitamin D in winter is adequate and safe for prevention of vitamin D deficiency in healthy children from Ushuaia, Tierra Del Fuego, Argentina. The Journal of steroid biochemistry and molecular b | 2007 | Central & South America | 18 | Healthy/Subclinical VDD | High | not stated | 100000 D2 oral q3months | 25OHD, Blood calcium, Phosphate, ALKP, Anthropometric measures |
| Soliman, A. Calcium homeostasis in 40 adolescents with beta-thalassemia major: a case-control study of the effects of intramuscular injection of a megadose of cholecalciferol. Pediatric Endocrinology Reviews | 2008 | Middle East | 40 | Other, beta-Thalassemia | High | 12-19 years | 10,000 U/Kg D3 IM once | 25OHD, Blood calcium, Phosphate, Clinical Rickets, Bone mass marker, ALKP, PTH, other - (see detailed form) |
|  | 2008 | Middle East | 26 | Healthy/Subclinical VDD | High | 12-19 years | 10000 U/Kg D3 IM once | 25OHD, Blood calcium, Phosphate, ALKP, PTH, other - (see detailed form) |
| Kislal, FM. Effect of different doses of vitamin D on osteocalcin and deoxypyridinoline in preterm infants.. Pediatrics International | 2008 | Europe | 11 | Premature and/or low birth weight | Medium/Unclear | 0-1 months | 200 IU/kg D2/3 oral Daily | Blood calcium, Phosphate, Bone mass marker, ALKP, other - (see detailed form) |
|  | 2008 | Europe | 15 | Premature and/or low birth weight | Medium/Unclear | 0-1 months | 400 IU/kg D2/3 oral Daily | Blood calcium, Phosphate, Bone mass marker, ALKP, other - (see detailed form) |
|  | 2008 | Europe | 11 | Premature and/or low birth weight | Medium/Unclear | 0-1 months | 800 IU/kg D2/3 oral Daily | Blood calcium, Phosphate, Bone mass marker, ALKP, other - (see detailed form) |
| Andersen, R. Effect of vitamin D supplementation on bone and vitamin D status among Pakistani immigrants in Denmark: a randomised double-blinded placebo-controlled intervention study.. British Journal of Nutrition | 2008 | Europe | 9 | Healthy/Subclinical VDD | Low | 10-15 years | 800 D3 oral Daily | 25OHD, Blood calcium, Urine calcium, Bone mass marker, PTH, other - (see detailed form) |
| Soliman, AT. Linear growth in relation to the circulating concentrations of insulin-like growth factor I, parathyroid hormone, and 25-hydroxy vitamin D in children with nutritional rickets before and after treatment: endocrine adaptation to vitamin D | 2008 | Middle East | 46 | Rickets | High | 0-3.99 year | 300000 D3 IM once | 25OHD, Blood calcium, Clinical Rickets, PTH |
| Hillman, LS. Percent true calcium absorption, mineral metabolism, and bone mass in children with arthritis: effect of supplementation with vitamin D3 and calcium. Arthritis Rheumatism | 2008 | North America | 16 | Other, Juvenile arthritis | Medium/Unclear | 3-15 years | 2000 D3 oral Daily | 25OHD , 125(OH)2D, Blood calcium, Urine calcium, Phosphate, Bone mass marker, Calcium absorption, other - (see detailed form) |
|  | 2008 | North America | 16 | Other, Juvenile arthritis | Medium/Unclear | 3-15 years | 2000 D3 oral Daily + Calcium | 25OHD , 125(OH)2D, Blood calcium, Urine calcium, Phosphate, Bone mass marker, Calcium absorption, other - (see detailed form) |
| Hillman, LS. Percent true calcium absorption, mineral metabolism, and bone mineralization in children with cystic fibrosis: effect of supplementation with vitamin D and calcium. Pediatric Pulmonology | 2008 | North America | 12 | Cystic fibrosis | Medium/Unclear | 3-15 years | 2000 D3 oral Daily | 25OHD , 125(OH)2D, Blood calcium, Urine calcium, Phosphate, Bone mass marker, PTH, Calcium absorption, other - (see detailed form) |
|  | 2008 | North America | 11 | Cystic fibrosis | Medium/Unclear | 3-15 years | 2000 D3 oral Daily + Calcium | 25OHD , 125(OH)2D, Blood calcium, Urine calcium, Phosphate, Bone mass marker, PTH, Calcium absorption, other - (see detailed form) |
| Sidbury, R. Randomized controlled trial of vitamin D supplementation for winter-related atopic dermatitis in Boston: a pilot study.. British Journal of Dermatology | 2008 | North America | 5 | Other, Atopic dermatitis | Medium/Unclear | not stated | 1000 D2 oral Daily | other - (see detailed form) |
| Maalouf, J. Short- and long-term safety of weekly high-dose vitamin D3 supplementation in school children. The Journal of Clinical Endocrinology and Metabolis | 2008 | Middle East | 8 | Healthy/Subclinical VDD | Medium/Unclear | 10-17 years | 14000 D3 (Vigantol) oral Weekly for 8 weeks | 25OHD , 125(OH)2D, Blood calcium, Adverse effects |
|  | 2008 | Middle East | 114 | Healthy/Subclinical VDD | Medium/Unclear | 10-17 years | 1400 D3 (Vigantol) oral Weekly for 1 year | 25OHD , 125(OH)2D, Blood calcium, Phosphate, ALKP, Adverse effects, other - (see detailed form) |
|  | 2008 | Middle East | 9 | Healthy/Subclinical VDD | Medium/Unclear | 10-17 years | 14000 D3 (Sigma crystallin) oral Weekly for 8 weeks | 25OHD , 125(OH)2D, Blood calcium, Adverse effects |
|  | 2008 | Middle East | 115 | Healthy/Subclinical VDD | Medium/Unclear | 10-17 years | 14000 D3 (Vigantol) oral Weekly for 1 year | 25OHD , 125(OH)2D, Blood calcium, Phosphate, ALKP, Adverse effects, other - (see detailed form) |
| Gordon, CM. Treatment of hypovitaminosis D in infants and toddlers. The Journal of Clinical Endocrinology and Metabolis | 2008 | North America | 12 | Healthy/Subclinical VDD | Medium/Unclear | 8-24 months | 2000 D2 oral Daily | 25OHD, Blood calcium, ALKP, PTH, other - (see detailed form) |
|  | 2008 | North America | 14 | Healthy/Subclinical VDD | Medium/Unclear | 8-24 months | 50000 D2 oral Weekly | 25OHD, Blood calcium, ALKP, PTH, other - (see detailed form) |
|  | 2008 | North America | 14 | Healthy/Subclinical VDD | Medium/Unclear | 8-24 months | 2000 D3 oral Daily | 25OHD, Blood calcium, ALKP, PTH, other - (see detailed form) |
| Billoo, AG. Comparison of oral versus injectable vitamin-D for the treatment of nutritional vitamin-D deficiency rickets. Journal of the College of Physicians and Surgeons - | 2009 | East Asia | 50 | Rickets | Medium/Unclear | 0.5-3 years | 200000 D3 oral once | Blood calcium, Phosphate, Clinical Rickets, ALKP, Hematology |
|  | 2009 | East Asia | 50 | Rickets | Medium/Unclear | 0.5-3 years | 200000 D3 IM once | Blood calcium, Phosphate, Clinical Rickets, ALKP, Hematology |
| Arpadi, SM. Effect of bimonthly supplementation with oral cholecalciferol on serum 25-hydroxyvitamin D concentrations in HIV-infected children and adolescents.. Pediatrics | 2009 | North America | 29 | HIV | Low | 6-16 years | 100000 D3 oral q2months | 25OHD, Blood calcium, Urine calcium, Anthropometric measures, Immune/Inflammatory marker |
| Thacher, TD. Meals and dephytinization affect calcium and zinc absorption in Nigerian children with rickets. The Journal of Nutrition | 2009 | Africa | 19 | Rickets | High | 2-10 years | 50000 D2 oral Weekly | 25OHD, Blood calcium, Phosphate, ALKP, PTH, Calcium absorption, other - (see detailed form) |
|  | 2009 | Africa | 15 | Healthy/Subclinical VDD | High | 2-10 years | 50000 D2 oral Weekly | 25OHD, Blood calcium, Phosphate, ALKP, PTH, Calcium absorption, other - (see detailed form) |
| Majak, P. The effect of oral steroids with and without vitamin D3 on early efficacy of immunotherapy in asthmatic children. Clinical & Experimental Allergy | 2009 | Europe | 18 | Asthma | Medium/Unclear | 6-12 years | 1000 D3 oral Weekly | 25OHD, Respiratory marker, Immune/Inflammatory marker |
| Thacher, TD. The effect of vitamin D2 and vitamin D3 on intestinal calcium absorption in Nigerian children with rickets. The Journal of Clinical Endocrinology and Metabolis | 2009 | Africa | 9 | Rickets | Medium/Unclear | 24-144 mont | 50000 D2 oral once | 25OHD , 125(OH)2D, Blood calcium, Urine calcium, Phosphate, ALKP, Calcium absorption, other - (see detailed form) |
|  | 2009 | Africa | 8 | Rickets | Medium/Unclear | 24-144 mont | 50000 D3 oral once | 25OHD , 125(OH)2D, Blood calcium, Urine calcium, Phosphate, ALKP, Calcium absorption, other - (see detailed form) |
| Shajari, A. Urinary calcium/creatinin ratio with different dosages of vitamin D3 prophylaxis in infants. Iranian Journal of Pediatrics | 2009 | Middle East | 30 | Healthy/Subclinical VDD | Medium/Unclear | 15-15 days | 50000 D3 oral Intermittent | 25OHD, Urine calcium |
| Boas, SR. Very high-dose ergocalciferol is effective for correcting vitamin D deficiency in children and young adults with cystic fibrosis. Journal of Cystic Fibrosis | 2009 | North America | 6 | Cystic fibrosis | High | not stated | 50000 D3 oral Daily | 25OHD, Respiratory marker |
| Arabi, A. Vitamin D receptor gene polymorphisms modulate the skeletal response to vitamin D supplementation in healthy girls. Bone | 2009 | Middle East | 58 | Healthy/Subclinical VDD | Medium/Unclear | 10-17 years | 1400 D3 oral Weekly | 25OHD , 125(OH)2D, Blood calcium, Phosphate, Clinical Rickets, Bone mass marker |
|  | 2009 | Middle East | 55 | Healthy/Subclinical VDD | Medium/Unclear | 10-17 years | 14000 D3 oral Weekly | 25OHD , 125(OH)2D, Blood calcium, Phosphate, Clinical Rickets, Bone mass marker |
| Dong, Y. A 16-week randomized clinical trial of 2000 international units daily vitamin D3 supplementation in black youth: 25-hydroxyvitamin D, adiposity, and arterial stiffness. The Journal of Clinical Endocrinology and Metabolis | 2010 | North America | 25 | Healthy/Subclinical VDD | Low | 14-18 years | 2000 D3 oral Daily | 25OHD, Blood calcium, Urine calcium, Cardiovascular marker, other - (see detailed form) |
| Ward, KA. A randomized, controlled trial of vitamin D supplementation upon musculoskeletal health in postmenarchal females. The Journal of Clinical Endocrinology and Metabolis | 2010 | Europe | 37 | Healthy/Subclinical VDD | Low | 12-14 years | 150000 D2 oral q3months | 25OHD, Blood calcium, Phosphate, Bone mass marker, PTH, Anthropometric measures, Renal marker |
| Mallet, E. Administration of a single Winter oral dose of 200,000 IU of vitamin D3 in adolescents in Normandy: evaluation of the safety and vitamin D status obtained. Archives de pÃ©diatrie | 2010 | Europe | 20 | Healthy/Subclinical VDD | High | not stated | 200000 D3 oral once (Measured after 2 weeks) | 25OHD, Blood calcium, Urine calcium, PTH |
|  | 2010 | Europe | 17 | Healthy/Subclinical VDD | High | not stated | 200000 D3 oral once (Measured after 3 months) | 25OHD, Blood calcium, Urine calcium, PTH |
| Bereket, A. Circulating insulin-like growth factor binding protein-4 (IGFBP-4) is not regulated by parathyroid hormone and vitamin D in vivo: evidence from children with rickets. Journal of Clinical Research in Pediatric Endocrino | 2010 | Middle East | 22 | Rickets | High | not stated | 300000 D2/3 oral once | 25OHD, Blood calcium, Phosphate, ALKP, PTH, other - (see detailed form) |
| Soliman, AT. Clinical responses to a mega-dose of vitamin D3 in infants and toddlers with vitamin D deficiency rickets. Journal of Tropical Pediatrics | 2010 | Middle East | 40 | Rickets | High | 0.1-3 years | 10000 IU/Kg D3 IM once | 25OHD, Blood calcium, Clinical Rickets, Bone mass marker, ALKP, PTH, Anthropometric measures |
| Shakiba, M. Combination of bolus dose vitamin D with routine vaccination in infants: a randomised trial. Singapore medical journal | 2010 | Middle East | 30 | Healthy/Subclinical VDD | Medium/Unclear | 15-30 days | 50000 D3 oral q2months | 25OHD, Blood calcium, Adverse effects |
| Thacher, TD. Comparison of metabolism of vitamins D2 and D3 in children with nutritional rickets. Journal of Bone and Mineral Research | 2010 | Africa | 12 | Rickets | High | not stated | 50000 D3 oral once | 25OHD , 125(OH)2D, Blood calcium, Phosphate, ALKP |
|  | 2010 | Africa | 12 | Healthy/Subclinical VDD | Medium/Unclear | not stated | 50000 D2 oral once | 25OHD , 125(OH)2D, Blood calcium, Phosphate, ALKP |
|  | 2010 | Africa | 11 | Healthy/Subclinical VDD | Medium/Unclear | not stated | 50000 D3 oral once | 25OHD , 125(OH)2D, Blood calcium, Phosphate, ALKP |
| Park, CY. Daily supplementation with 25 Î¼g cholecalciferol does not increase calcium absorption or skeletal retention in adolescent girls with low serum 25-hydroxyvitamin D. The Journal of Nutrition | 2010 | North America | 13 | Healthy/Subclinical VDD | High | 12-14 years | 1000 D3 oral Daily | 25OHD , 125(OH)2D, Blood calcium, Urine calcium, Phosphate, PTH, Calcium absorption, other - (see detailed form) |
| Ghazi, AA. Effects of different doses of oral cholecalciferol on serum 25(OH)D, PTH, calcium and bone markers during fall and winter in schoolchildren. European Journal of Clinical Nutrition | 2010 | Middle East | 70 | Healthy/Subclinical VDD | Low | 14-20 years | 50000 D3 oral Monthly | 25OHD, Blood calcium, Urine calcium, PTH |
|  | 2010 | Middle East | 70 | Healthy/Subclinical VDD | Low | 14-20 years | 50000 D3 oral q2months | 25OHD, Blood calcium, Urine calcium, PTH |
| Manaseki-Holland, S. Effects of vitamin D supplementation to children diagnosed with pneumonia in Kabul: a randomised controlled trial. Tropical Medicine & International Health | 2010 | East Asia | 224 | Pneumonia | Low | 1-36 months | 100000 D3 oral once | Respiratory marker, Immune/Inflammatory marker, Adverse effects, other - (see detailed form) |
| Marwaha, RK. Impact of two regimens of vitamin D supplementation on calcium - vitamin D - PTH axis of schoolgirls of Delhi. Indian Pediatrics | 2010 | East Asia | 141 | Healthy/Subclinical VDD | Medium/Unclear | 6-17 years | 60000 D3 oral q2months | 25OHD, Blood calcium, Phosphate, ALKP, PTH |
|  | 2010 | East Asia | 149 | Healthy/Subclinical VDD | Medium/Unclear | 6-17 years | 60000 D3 oral Monthly | 25OHD, Blood calcium, Phosphate, ALKP, PTH |
| Urashima, M. Randomized trial of vitamin D supplementation to prevent seasonal influenza A in schoolchildren.. American Journal of Clinical Nutrition | 2010 | East Asia | 217 | Healthy/Subclinical VDD | Low | 6-15 years | 1200 D3 oral Daily | Respiratory marker, Immune/Inflammatory marker, Adverse effects, other - (see detailed form) |
| Hari, P. Vitamin D insufficiency and effect of cholecalciferol in children with chronic kidney disease. Pediatric Nephrology | 2010 | East Asia | 42 | Renal disease | High | 1-16 years | 200000 D3 oral Daily | 25OHD, Blood calcium, Phosphate, ALKP, PTH, Renal marker, other - (see detailed form) |
| Khadikar, AV. Vitamin D supplementation and bone mass accrual in underprivileged adolescent Indian girls. Asia Pacific Journal of Clinical Nutrition | 2010 | East Asia | 25 | Healthy/Subclinical VDD | Medium/Unclear | 14-15 years | 300000 D2 oral q3months | 25OHD, Blood calcium, Phosphate, Bone mass marker, ALKP, PTH, Anthropometric measures |
| Ekbote, VH. A pilot randomized controlled trial of oral calcium and vitamin D supplementation using fortified laddoos in underprivileged Indian toddlers.. European Journal of Clinical Nutrition | 2011 | East Asia | 30 | Healthy/Subclinical VDD | Medium/Unclear | 2-3 years | 30000 D3 oral Monthly + Calcium (405 mg) | 25OHD, Blood calcium, Phosphate, Bone mass marker, ALKP, PTH |
|  | 2011 | East Asia | 30 | Healthy/Subclinical VDD | Medium/Unclear | 2-3 years | 30000 D3 oral Monthly + Calcium (156 mg) | 25OHD, Blood calcium, Phosphate, Bone mass marker, ALKP, PTH |
| Ashraf, AP. Associations of serum 25-hydroxyvitamin D and components of the metabolic syndrome in obese adolescent females. Obesity | 2011 | North America | 14 | Obesity | High | not stated | 50000 D2 oral Weekly | 25OHD, PTH, Cardiovascular marker, Diabetes marker, Immune/Inflammatory marker, other - (see detailed form) |
| Kumar, GT. Effect of weekly vitamin D supplements on mortality, morbidity, and growth of low birthweight term infants in India up to age 6 months: randomised controlled trial.. British Medical Journal | 2011 | East Asia | 1039 | Premature and/or low birth weight | Low | 0-2 days | 1400 D3 oral Weekly | 25OHD, Anthropometric measures, Adverse effects, other - (see detailed form) |
| Soliman, AT. Manifestations of severe vitamin D deficiency in adolescents: effects of intramuscular injection of a megadose of cholecalciferol. Journal of Tropical Pediatrics | 2011 | Middle East | 40 | Healthy/Subclinical VDD | High | 12-18 years | 10,000 IU/Kg D3 IM once | 25OHD, Blood calcium, Urine calcium, Phosphate, Clinical Rickets, PTH, Anthropometric measures, other - (see detailed form) |
| Alonso, A. Prophylactic vitamin D in healthy infants: assessing the need.. Metabolism | 2011 | Europe | 48 | Healthy/Subclinical VDD | Medium/Unclear | 0-15 days | 402 D3 oral Daily | 25OHD, PTH, Anthropometric measures |
| Siafarikas, A. Randomised controlled trial analysing supplementation with 250 versus 500 units of vitamin D3, sun exposure and surrounding factors in breastfed infants.. Archives of disease in childhood | 2011 | Europe | 20 | Healthy/Subclinical VDD | Low | 1-1 days | 500 D3 oral Daily | 25OHD, Blood calcium, Urine calcium, Phosphate, Clinical Rickets, ALKP |
| Rich-Edwards, JW. Randomized trial of fortified milk and supplements to raise 25-hydroxyvitamin D concentrations in schoolchildren in Mongolia. American Journal of Clinical Nutrition | 2011 | East Asia | 92 | Healthy/Subclinical VDD | Low | 9-11 years | 1950 D3 oral Daily | 25OHD |
| Shakiba, M. Study to evaluate two dosage regimens of vitamin D through an academic year in middle school girls: a randomized trial. Acta Medica Iranica | 2011 | Middle East | 34 | Healthy/Subclinical VDD | Medium/Unclear | 12-15 years | 50000 D3 oral q3months | 25OHD, Urine calcium |
|  | 2011 | Middle East | 34 | Healthy/Subclinical VDD | Medium/Unclear | 12-15 years | 100000 D3 oral q3months | 25OHD, Urine calcium |
| Shakinba, M. The optimal dose of vitamin D in growing girls during academic years: A randomized trial. Turkish Journal of Medical Sciences | 2011 | Middle East | 30 | Healthy/Subclinical VDD | Medium/Unclear | 12-15 years | D3 oral Intermittent: 300,000 IU at enrolment + 50,000 IU monthly | 25OHD |
|  | 2011 | Middle East | 30 | Healthy/Subclinical VDD | Medium/Unclear | 12-15 years | D3 oral Intermittent: 300,000 IU at enrolment + 100,000 IU every 3 months | 25OHD |
|  | 2011 | Middle East | 30 | Healthy/Subclinical VDD | Medium/Unclear | 12-15 years | 50000 D3 oral Monthly | 25OHD |
|  | 2011 | Middle East | 30 | Healthy/Subclinical VDD | Medium/Unclear | 12-15 years | 100000 D3 oral q3months | 25OHD |
| Kakalia, S. Vitamin D supplementation and CD4 count in children infected with human immunodeficiency virus. The Journal of Pediatrics | 2011 | North America | 18 | HIV | Medium/Unclear | 3-18 years | 5600 D3 oral Weekly | 25OHD , 125(OH)2D, Blood calcium, Urine calcium, PTH, Immune/Inflammatory marker |
|  | 2011 | North America | 18 | HIV | Medium/Unclear | 3-18 years | 11200 D3 oral Weekly | 25OHD , 125(OH)2D, Blood calcium, Urine calcium, PTH, Immune/Inflammatory marker |
| Hill, KM. Bone turnover is not influenced by serum 25-hydroxyvitamin D in pubertal healthy black and white children. Bone | 2012 | North America | 65 | Healthy/Subclinical VDD | Medium/Unclear | not stated | 1000 D3 oral Daily | 25OHD, Phosphate, Bone mass marker, ALKP, other - (see detailed form) |
|  | 2012 | North America | 64 | Healthy/Subclinical VDD | Medium/Unclear | not stated | 2000 D3 oral Daily | 25OHD, Phosphate, Bone mass marker, ALKP, other - (see detailed form) |
|  | 2012 | North America | 64 | Healthy/Subclinical VDD | Medium/Unclear | not stated | 4000 D3 oral Daily | 25OHD, Phosphate, Bone mass marker, ALKP, other - (see detailed form) |
| Soliman, A. Clinical, biochemical and radiological manifestations of severe vitamin d deficiency in adolescents versus children: response to therapy. Georgian Medical News | 2012 | Middle East | 45 | Healthy/Subclinical VDD | High | not stated | 10000 IU/Kg D3 IM once | 25OHD, Blood calcium, Urine calcium, Phosphate, Clinical Rickets, ALKP, PTH, Anthropometric measures, other - (see detailed form) |
|  | 2012 | Middle East | 36 | Healthy/Subclinical VDD | High | not stated | 10000 IU/Kg D3 IM once | 25OHD, Blood calcium, Urine calcium, Phosphate, Clinical Rickets, ALKP, PTH, Anthropometric measures, other - (see detailed form) |
| Arpadi, SM. Effect of supplementation with cholecalciferol and calcium on 2-y bone mass accrual in HIV-infected children and adolescents: a randomized clinical trial. American Journal of Clinical Nutrition | 2012 | North America | 30 | HIV | Medium/Unclear | not stated | 100000 D3 oral q2months | 25OHD, Blood calcium, Urine calcium, Bone mass marker |
| Kumar, GT. Effect of vitamin D supplementation of low birth weight term Indian infants from birth on cytokine production at 6 months.. European Journal of Clinical Nutrition | 2012 | East Asia | 1039 | Premature and/or low birth weight | Low | 0-2 days | 1400 D3 oral Weekly | 25OHD, Immune/Inflammatory marker |
| Manaseki-Holland, S. Effect on the incidence of pneumonia of vitamin D supplementation by quarterly bolus dose to infants in Kabul: a randomised controlled superiority trial. Lancet | 2012 | East Asia | 1524 | Healthy/Subclinical VDD | Low | 1-11 months | 100000 D3 oral q4months | 25OHD, Anthropometric measures, Respiratory marker, Adverse effects, other - (see detailed form) |
| Shneider, BL. Efficacy of fat-soluble vitamin supplementation in infants with biliary atresia.. Pediatrics | 2012 | North America | 92 | Malabsorption, Biliary atresia | High | 0-6 months | 800 D3 oral Daily | 25OHD, other - (see detailed form) |
| Shroff, R. Ergocalciferol supplementation in children with CKD delays the onset of secondary hyperparathyroidism: a randomized trial. Clinical Journal of The American Society of Nephrol | 2012 | Europe | 24 | Renal disease | Medium/Unclear | 0-17 years | 600-8000 D2 oral Daily | 25OHD , 125(OH)2D, Blood calcium, Phosphate, ALKP, PTH, Adverse effects |
| Osunkwo, L. High dose vitamin D therapy for chronic pain in children and adolescents with sickle cell disease: results of a randomized double blind pilot study. British Journal of Haematology | 2012 | North America | 20 | Other, Sickle cell | Medium/Unclear | 7-21 years | D3 oral Weekly: 40000 to 100000/week + 200 IU/day in daily supplement (20.0-20.9kg:40000IU/wk; 30.0-30.9kg:60000IU/wk; 40.0-40.9kg:80000/wk; >50kg:100000IU/wk) | 25OHD, other - (see detailed form) |
| Holmlund-Suila, E. High-dose vitamin d intervention in infants--effects on vitamin d status, calcium homeostasis, and bone strength. The Journal of Clinical Endocrinology and Metabolis | 2012 | Europe | 38 | Healthy/Subclinical VDD | Low | 0-0 days | 1200 D3 oral Daily | 25OHD, Blood calcium, Urine calcium, Phosphate, Bone mass marker, PTH, Anthropometric measures, Hematology, Immune/Inflammatory marker |
|  | 2012 | Europe | 37 | Healthy/Subclinical VDD | Low | 0-0 days | 1600 D3 oral Daily | 25OHD, Blood calcium, Urine calcium, Phosphate, Bone mass marker, PTH, Anthropometric measures, Hematology, Immune/Inflammatory marker |
| Carnes, J. Intermittent high-dose vitamin D corrects vitamin D deficiency in adolescents: a pilot study. European Journal of Clinical Nutrition | 2012 | Australia/New Zealand | 7 | Healthy/Subclinical VDD | Medium/Unclear | 15-17 years | 150000 D3 oral q6months | 25OHD, Blood calcium |
|  | 2012 | Australia/New Zealand | 7 | Healthy/Subclinical VDD | Medium/Unclear | 15-17 years | 300000 D3 oral q6months | 25OHD, Blood calcium |
| Dogan, M. Oxidant/antioxidant system markers and trace element levels in children with nutritional rickets. Journal of Pediatric Endocrinology & Metabolism | 2012 | Middle East | 30 | Rickets | High | 0.25-15 yea | 300000 D2/3 IM once | 25OHD, Blood calcium, Phosphate, ALKP, PTH, Immune/Inflammatory marker, other - (see detailed form) |
| Lewis, E. Relationship of 25-hydroxyvitamin D and asthma control in children. Annals of Allergy, Asthma, & Immunology | 2012 | North America | 15 | Asthma | Medium/Unclear | 6-17 years | 1000 D3 oral Daily | 25OHD, Respiratory marker |
| Castaneda, RA. Response to vitamin D3 supplementation in obese and non-obese Caucasian adolescents. Hormone Research In Paediatrics | 2012 | North America | 22 | Healthy/Subclinical VDD | High | 12-18 years | 2000 D3 oral Daily | 25OHD, Blood calcium, Phosphate, PTH, Anthropometric measures |
|  | 2012 | North America | 27 | Obesity | High | 12-18 years | 2000 D3 oral Daily | 25OHD, Blood calcium, Phosphate, PTH, Anthropometric measures |
| Soliman, AT. The effect of vitamin D therapy on hematological indices in children with vitamin D deficiency. The Journal of Tropical Pediatrics | 2012 | Middle East | 40 | Healthy/Subclinical VDD | High | not stated | 10000 IU/Kg D3 IM once | 25OHD, Cardiovascular marker, Hematology, Immune/Inflammatory marker, other - (see detailed form) |
| Emel, T. Therapy strategies in vitamin D deficiency with or without rickets: efficiency of low-dose stoss therapy. Journal of Pediatric Endocrinology & Metabolism | 2012 | Middle East | 21 | Rickets | Medium/Unclear | not stated | 2000 D3 oral Daily | 25OHD, Blood calcium, Urine calcium, PTH |
|  | 2012 | Middle East | 21 | Rickets | Medium/Unclear | not stated | 150000 D3 oral once | 25OHD, Blood calcium, Urine calcium, PTH |
| Choudhary, N. Vitamin d supplementation for severe pneumonia a randomized controlled trial.. Indian pediatrics | 2012 | East Asia | 100 | Pneumonia | Low | 2-60 months | D2/3 oral Daily: 1000 (age < 1 year), 2000 IU (age 1-5 years) | Respiratory marker, Adverse effects, other - (see detailed form) |
| Shedeed, SA. Vitamin D supplementation in infants with chronic congestive heart failure. Pediatric Cardiology | 2012 | Middle East | 42 | Other, Congestive heart failure | Low | not stated | 1000 D3 oral Daily | 25OHD, Blood calcium, Phosphate, PTH, Cardiovascular marker, Immune/Inflammatory marker |
| Ganmaa, D. Vitamin D, tuberculin skin test conversion, and latent tuberculosis in Mongolian school-age children: a randomized, double-blind, placebo-controlled feasibility trial.. American Journal of Clinical Nutrition | 2012 | East Asia | 61 | Healthy/Subclinical VDD | Low | 12-15 years | 800 D3 oral Daily | 25OHD, Immune/Inflammatory marker |
| Putman, MS. A randomized clinical trial of vitamin d supplementation in healthy adolescents. Journal of Adolescent Health | 2013 | North America | 29 | Healthy/Subclinical VDD | Medium/Unclear | 11-19 years | 1000 D3 oral Daily | 25OHD, Blood calcium, Phosphate, Bone mass marker, ALKP, PTH, Diabetes marker, other - (see detailed form) |
| Lewis, RD. A Randomized Trial of Vitamin D3 Supplementation in Children: Dose-Response Effects on Vitamin D Metabolites and Calcium Absorption. The Journal of Clinical Endocrinology and Metabolis | 2013 | North America | 65 | Healthy/Subclinical VDD | Low | 9-13 years | 1000 D3 oral Daily | 25OHD , 125(OH)2D, Blood calcium, Urine calcium, PTH, Calcium absorption |
|  | 2013 | North America | 64 | Healthy/Subclinical VDD | Low | 9-13 years | 2000 D3 oral Daily | 25OHD , 125(OH)2D, Blood calcium, Urine calcium, PTH, Calcium absorption |
|  | 2013 | North America | 64 | Healthy/Subclinical VDD | Low | 9-13 years | 4000 D3 oral Daily | 25OHD , 125(OH)2D, Blood calcium, Urine calcium, PTH, Calcium absorption |
| Belenchia, AM. Correcting vitamin D insufficiency improves insulin sensitivity in obese adolescents: a randomized controlled trial. American Journal of Clinical Nutrition | 2013 | North America | 21 | Obesity | Low | 9-19 years | 4000 D3 oral Daily | 25OHD, Immune/Inflammatory marker |
| Gallo, S. Effect of different dosages of oral vitamin D supplementation on vitamin D status in healthy, breastfed infants. The Journal of the American Medical Association | 2013 | North America | 39 | Healthy/Subclinical VDD | Low | 0-1 months | 800 D3 oral Daily | 25OHD, Blood calcium, Urine calcium, Phosphate, Bone mass marker, ALKP, Anthropometric measures |
|  | 2013 | North America | 38 | Healthy/Subclinical VDD | Low | 0-1 months | 1200 D3 oral Daily | 25OHD, Blood calcium, Urine calcium, Phosphate, Bone mass marker, ALKP, Anthropometric measures |
|  | 2013 | North America | 16 | Healthy/Subclinical VDD | Low | 0-1 months | 1600 D3 oral Daily | 25OHD, Blood calcium, Urine calcium, Phosphate, Bone mass marker, ALKP, Anthropometric measures |
| Yadav, M. Effect of Vitamin D Supplementation on Moderate to Severe Bronchial Asthma.. Indian J Pediatrics | 2013 | East Asia | 50 | Asthma | Low | 3-14 years | 60000 D3 oral Monthly | Respiratory marker, Adverse effects, other - (see detailed form) |
| Poomthavorn, P. Effects of correction of vitamin D insufficiency on serum osteocalcin and glucose metabolism in obese children. Clinical Endocrinology | 2013 | East Asia | 72 | Obesity | High | 6-18 years | 20000 D2 oral Daily | 25OHD, Bone mass marker, PTH, Anthropometric measures, Diabetes marker, other - (see detailed form) |
| Kelishadi, R. Effects of vitamin D supplementation on insulin resistance and cardiometabolic risk factors in children with metabolic syndrome: a triple-masked controlled trial. Jornal de Pediatria | 2013 | Middle East | 25 | Obesity | Low | 10-16 years | 50000 D3 oral Weekly | 25OHD, Cardiovascular marker, Diabetes marker, other - (see detailed form) |
| Garg, MK. Efficacy of vitamin D loading doses on serum 25-hydroxy vitamin D levels in school going adolescents: an open label, non-randomized prospective trial. Journal of Pediatric Endocrinology & Metabolism | 2013 | East Asia | 238 | Healthy/Subclinical VDD | Medium/Unclear | 10-15 years | 60000 D3 oral Weekly (4 weeks) | 25OHD, Blood calcium, Urine calcium, Phosphate, ALKP |
|  | 2013 | East Asia | 139 | Healthy/Subclinical VDD | Medium/Unclear | 10-15 years | 60000 D3 oral Weekly (6 weeks) | 25OHD, Blood calcium, Urine calcium, Phosphate, ALKP |
|  | 2013 | East Asia | 134 | Healthy/Subclinical VDD | Medium/Unclear | 10-15 years | 60000 D3 oral Weekly (8 weeks) | 25OHD, Blood calcium, Urine calcium, Phosphate, ALKP |
| Ekbote, VH. Enhanced effect of zinc and calcium supplementation on bone status in growth hormone-deficient children treated with growth hormone: a pilot randomized controlled trial.. Endocrine | 2013 | East Asia | 15 | Other, Growth hormone deficiency | Medium/Unclear | not stated | 60000 D3 oral q3months | 25OHD, Blood calcium, Bone mass marker, PTH, other - (see detailed form) |
|  | 2013 | East Asia | 16 | Other, Growth hormone deficiency | Medium/Unclear | not stated | 60000 D3 oral q3months + Zinc | 25OHD, Blood calcium, Bone mass marker, PTH, other - (see detailed form) |
| Rianthavorn, P. Ergocalciferol decreases erythropoietin resistance in children with chronic kidney disease stage 5. Pediatric Nephrology | 2013 | East Asia | 10 | Renal disease | Medium/Unclear | 0-18 years | D2 oral Intermittent: Severe 25OHD deficiency (<5 ng/mL): 40000 IU/week for 4 weeks followed by 40000 IU biweekly for 8 weeks. Mild 25OHD deficiency (levels 5-15 ng/mL): 40000 IU biweekly for 12 weeks. 25OHD insufficiency (25OHD 16-30 ng/mL): 40000 IU/4weeks for 12 weeks | 25OHD, Blood calcium, PTH, Renal marker, other - (see detailed form) |
| Principi, N. Impact of vitamin D administration on immunogenicity of trivalent inactivated influenza vaccine in previously unvaccinated children. Human Vaccines & Immunotherapeutics | 2013 | Europe | 59 | Recurrent acute otitis media | Low | 2-5 years | 1000 D3 oral Daily | 25OHD, Immune/Inflammatory marker, Adverse effects |
| Khadgawat, R. Impact of vitamin D fortified milk supplementation on vitamin D status of healthy school children aged 10-14 years. Osteoporosis International | 2013 | East Asia | 258 | Healthy/Subclinical VDD | Low | 10-14 years | 1000 D3 oral Daily | 25OHD, Blood calcium, Urine calcium, Phosphate, ALKP, other - (see detailed form) |
| Kari, JA. Is high-dose cholecalciferol justified in children with chronic kidney disease who failed low-dose maintenance therapy?. Pediatric Nephrology | 2013 | Middle East | 19 | Renal disease | High | not stated | 300000 D3 IM once | 25OHD, Blood calcium, Phosphate, PTH |
| Aggarwal, V. Management of nutritional rickets in Indian children: a randomized controlled trial. Journal of Tropical Pediatrics | 2013 | East Asia | 23 | Rickets | Medium/Unclear | 0.5-5 years | 600000 D2/3 IM once | 25OHD, Blood calcium, Phosphate, Clinical Rickets, Bone mass marker, ALKP, PTH |
|  | 2013 | East Asia | 22 | Rickets | Medium/Unclear | 0.5-5 years | 600000 D2/3 IM once + Calcium | 25OHD, Blood calcium, Phosphate, Clinical Rickets, Bone mass marker, ALKP, PTH |
| Cayir, A. Serum vitamin D levels in children with recurrent otitis media. European Archives of Oto-Rhino-Laryngology | 2013 | Middle East | 84 | Recurrent acute otitis media | High | 1-5 years | D3 oral Daily: 5000 (25OHD < 15) or 400 (25OHD > 15) | 25OHD, PTH, Adverse effects, other - (see detailed form) |
|  | 2013 | Middle East | 109 | Healthy/Subclinical VDD | High | 1-5 years | D3 oral Daily: 5000 (25OHD < 15) or 400 (25OHD > 15) | 25OHD, PTH, Adverse effects, other - (see detailed form) |
| Shepherd, D. Single high-dose oral vitamin D3 (stoss) therapy--a solution to vitamin D deficiency in children with cystic fibrosis?. Journal of Cystic Fibrosis | 2013 | Australia/New Zealand | 42 | Cystic fibrosis | High | not stated | Depends on D3 oral once | 25OHD, Blood calcium |
| Abrams, S. Supplementation with 1000 IU vitamin D/d leads to parathyroid hormone suppression, but not increased fractional calcium absorption, in 4-8-y-old children: a double-blind randomized controlled trial. American Journal of Clinical Nutrition | 2013 | North America | 32 | Healthy/Subclinical VDD | Low | 4-9 years | 1000 D2 oral Daily | 25OHD, Urine calcium, PTH, Adverse effects, Calcium absorption |
| Marchisio, P. Vitamin D Supplementation Reduces the Risk of Acute Otitis Media in Otitis-prone Children. The Pediatric Infectious Disease Journal | 2013 | Europe | 58 | Recurrent acute otitis media | Low | 1-5 years | 1000 D3 oral Daily | 25OHD, Immune/Inflammatory marker, Adverse effects, other - (see detailed form) |
| Aluisio, AR. Vitamin D3 Supplementation and Childhood Diarrhea: A Randomized Controlled Trial. Pediatrics | 2013 | East Asia | 1524 | Healthy/Subclinical VDD | Low | 1-11 months | 100000 D3 oral q4months | 25OHD, Respiratory marker, Adverse effects, other - (see detailed form) |
| Wingate, KE. 25-Hydroxyvitamin D concentrations in children with Crohn's disease supplemented with either 2000 or 400 IU daily for 6 months: a randomized controlled study. Journal of Pediatrics | 2014 | North America | 43 | Malabsorption, Crohn's disease | Low | 8-18 years | 2000 D3 oral Daily | 25OHD, Blood calcium, Urine calcium, Phosphate, Immune/Inflammatory marker, other - (see detailed form) |
| Mittal, H. 300,000 IU or 600,000 IU of oral vitamin D3 for treatment of nutritional rickets: a randomized controlled trial. Indian Pediatrics | 2014 | East Asia | 38 | Rickets | Low | 0.5-6 years | 300000 D3 oral once | 25OHD, Blood calcium, Urine calcium, Clinical Rickets, PTH |
|  | 2014 | East Asia | 38 | Rickets | Low | 0.5-6 years | 600000 D3 oral once | 25OHD, Blood calcium, Urine calcium, Clinical Rickets, PTH |
| Mondal, K. A Randomized controlled trial on safety and efficacy of single intramuscular versus staggered oral dose of 600 000IU Vitamin D in treatment of nutritional rickets. Journal of Tropical Pediatrics | 2014 | East Asia | 37 | Rickets | Low | 0.5-5 years | 600000 D3 IM once | 25OHD, Blood calcium, Urine calcium, Clinical Rickets |
|  | 2014 | East Asia | 34 | Rickets | Low | 0.5-5 years | 60000 D3 oral Weekly | 25OHD, Blood calcium, Urine calcium, Clinical Rickets |
| Shakiba, M. Comparison of two regimens of vitamin D supplementation for vitamin D-deficient neonates. Singapore Medical Journal | 2014 | Middle East | 34 | Healthy/Subclinical VDD | High | 0-15 days | 30000 D3 oral Monthly | 25OHD |
| Nader, NS. Effect of vitamin D3 supplementation on serum 25(OH)D, lipids and markers of insulin resistance in obese adolescents: a prospective, randomized, placebo-controlled pilot trial. Hormone research in pdiatrics | 2014 | North America | 28 | Obesity | Low | 12-18 years | 2000 D3 oral Daily | 25OHD, Blood calcium, PTH, Cardiovascular marker, Immune/Inflammatory marker |
| Pappa, HM. Maintenance of optimal vitamin D status in children and adolescents with inflammatory bowel disease: a randomized clinical trial comparing two regimens. Journal of Clinical Endocrinology & Metabolism | 2014 | North America | 31 | Malabsorption, Inflammatory bowel diseas | Low | 5-21 years | 1000 IU in D2 oral Daily | 25OHD, Blood calcium, Urine calcium, Phosphate, PTH, Immune/Inflammatory marker, other - (see detailed form) |
| Taheri, PA. Prophylactic effect of low dose vitamin D in osteopenia of prematurity: a clinical trial study. Acta Medica Iranica | 2014 | Middle East | 30 | Premature and/or low birth weight | Medium/Unclear | 0-0 days | 200 D3 oral Daily | 25OHD, Blood calcium, Clinical Rickets, Bone mass marker |
|  | 2014 | Middle East | 30 | Premature and/or low birth weight | Medium/Unclear | 0-0 days | 400 D3 oral Daily | 25OHD, Blood calcium, Clinical Rickets, Bone mass marker |
| Camargo, CA Jr.. Randomized trial of vitamin D supplementation for winter-related atopic dermatitis in children. Journal of Allergy & Clinical Immunology | 2014 | East Asia | 58 | Other, Atopic dermatitis | Medium/Unclear | 2-17 years | 1000 D3 oral Daily | other - (see detailed form) |
| Natarajan, CK. Trial of daily vitamin D supplementation in preterm infants. Pediatrics | 2014 | East Asia | 48 | Premature and/or low birth weight | Low | 0-14 days | 800 D3 oral Daily | 25OHD, Blood calcium, Urine calcium, Phosphate, Bone mass marker, ALKP, PTH |
|  | 2014 | East Asia | 48 | Premature and/or low birth weight | Low | 0-14 days | 400 D3 oral Daily | 25OHD, Blood calcium, Urine calcium, Phosphate, Bone mass marker, ALKP, PTH |
| Baris, S. Vitamin D as an adjunct to subcutaneous allergen immunotherapy in asthmatic children sensitized to house dust mite.. Allergy | 2014 | Middle East | 18 | Asthma | Low | 5-15 years | 650 D3 oral Daily | 25OHD, Blood calcium, Urine calcium, PTH, Respiratory marker, Immune/Inflammatory marker, other - (see detailed form) |
| Grant, CC. Vitamin d during pregnancy and infancy and infant serum 25-hydroxyvitamin d concentration.. Pediatrics | 2014 | Australia/New Zealand | 85 | Healthy/Subclinical VDD | Low | 0-0 days | 800 D3 oral Daily | 25OHD, Blood calcium |
| Tergestina, M. Vitamin D status and adequacy of standard supplementation in preterm neonates from South India. Journal of Pediatric Gastroenterology & Nutrition | 2014 | East Asia | 90 | Premature and/or low birth weight | High | 0-0 days | 400 D3 oral Daily | 25OHD, Blood calcium, PTH |
| Ziegler, EE. Vitamin D supplementation of breastfed infants: a randomized dose-response trial. Pediatric Research | 2014 | North America | 56 | Healthy/Subclinical VDD | Low | 28-28 days | 600 D3 oral Daily | 25OHD, Blood calcium, PTH |
|  | 2014 | North America | 41 | Healthy/Subclinical VDD | Low | 28-28 days | 800 D3 oral Daily | 25OHD, Blood calcium, PTH |
| Thacher, TD. Vitamin D treatment in calcium-deficiency rickets: a randomised controlled trial. Archives of Disease in Childhood | 2014 | Africa | 44 | Rickets | Low | not stated | 50000 D2 oral Monthly | 25OHD, Blood calcium, Clinical Rickets, Bone mass marker, ALKP |
| Al-Shaar, L. Vitamin D3 dose requirement to raise 25-hydroxyvitamin D to desirable levels in adolescents: results from a randomized controlled trial. Journal of Bone & Mineral Research | 2014 | Middle East | 121 | Healthy/Subclinical VDD | Low | 10-17 years | 1400 D3 oral Weekly | 25OHD |
|  | 2014 | Middle East | 115 | Healthy/Subclinical VDD | Low | 10-17 years | 14000 D3 oral Weekly | 25OHD |
| Javed, A. Cholecalciferol Supplementation Does Not Influence beta-Cell Function and Insulin Action in Obese Adolescents: A Prospective Double-Blind Randomized Trial. Journal of Nutrition | 2015 | North America | 26 | Obesity | Low | 12-18 years | 2000 D3 oral Daily | 25OHD, Anthropometric measures, Cardiovascular marker, Diabetes marker, other - (see detailed form) |
