# Supplementary material for: A systematic review of pediatric clinical trials of high dose vitamin D
Source: PeerJ. 2016 Feb 25;4:e1701. doi: 10.7717/peerj.1701 (PMC4782742; doi:10.7717/peerj.1701)
Supplement: Appendix S1 [file peerj-04-1701-s011.doc]

Medline

| 1. exp Vitamin d/ |
| --- |
| 2. (vitamin adj (d or d2 or d3)).tw. |
| 3. Calcifediol/ |
| 4. calcidiol.tw. |
| 5. Ergocalciferols/ |
| 6. Ergocalciferol$.tw. |
| 7. Cholecalciferol/ |
| 8. Cholecalciferol$.tw. |
| 9. calciferol.tw. |
| 10. Vitamin D Deficiency/dh, dt |
| 11. or/1-10 |
| 12. (25-hydroxyvitamin D or 25-hydroxy vitamin d or Plasma vitamin D).tw. |
| 13. 64719-49-9.rn. |
| 14. 25OHD3.tw. |
| 15. "25(OH)D3".tw. |
| 16. 25-OHD3.tw. |
| 17. "25-(OH)D3".tw. |
| 18. 25OHD.tw. |
| 19. "25(OH)D".tw. |
| 20. 25-OHD.tw. |
| 21. "25-(OH)D".tw. |
| 22. (25-hydroxycholecalciferol or 25-hydroxyergocalciferol).tw. |
| 23. Calcium/bl, ur |
| 24. plasma calcidiol.tw. |
| 25. (Urine calcium or (calcium adj3 ratio)).tw. |
| 26. or/12-25 |
| 27. exp Vitamin D Deficiency/ not Vitamin D Deficiency/dh, dt |
| 28. (avitaminosis and (d or d2 or d3)).tw. |
| 29. Vitamin D/to |
| 30. No-Observed-Adverse-Effect Level/ |
| 31. upper limit$.tw. |
| 32. UL.tw. |
| 33. (excess$ or toxic$).tw. |
| 34. (noael or noel).tw. |
| 35. (no observed adj2 effect$).tw. |
| 36. Calcification, Physiologic/de |
| 37. Hypercalcemia/ |
| 38. Kidney Calculi/ |
| 39. Nephrocalcinosis/ |
| 40. Urinary Calculi/ |
| 41. Bladder Calculi/ |
| 42. Ureteral Calculi/ |
| 43. Calcinosis/ |
| 44. Hypercalcemi$.tw. |
| 45. (Burnett$ adj2 syndrome$).tw. |
| 46. Hypercalciuri$.tw. |
| 47. exp Vitamin d/ae or Calcifediol/ae or Ergocalciferols/ae or Cholecalciferol/ae |
| 48. (Side effect* or adverse effect$).tw. |
| 49. or/27-48 |
| 50. 11 and (26 or 49) |
| 51. ((randomized controlled trial or controlled clinical trial).pt. or randomized.ab. or placebo.ab. or clinical trials as topic.sh. or randomly.ab. or trial.ti.) not (exp animals/ not humans.sh.) |
| 52. (Single arm or pilot or cross-over or n-of-1).tw. |
| 53. Double-blind Method/ or Single-blind Method/ |
| 54. (clin$ adj25 trial$).ti,ab. |
| 55. ((singl$ or doubl$ or trebl$ or tripl$) adj25 (blind$ or mask$)).ti,ab. |
| 56. Placebos/ |
| 57. 50 and (or/51-56) |
| 58. limit 50 to clinical trial, all |
| 59. or/57-58 |
| 60. ((single adj2 dose) or bolus or stoss* or single day or mega*).tw. |
| 61. Dose-Response Relationship, Drug/ |
| 62. 60 or 61 |
| 63. 11 and 62 |
| 64. 59 or 63 |
| 65. 64 and (child* or adolescent or infan*).mp. |
| 66. 64 and ((Infan* or newborn* or new-born* or perinat* or neonat* or baby or baby* or babies or toddler* or minors or minors* or boy or boys or boyfriend or boyhood or girl* or kid or kids or child or child* or children* or schoolchild* or schoolchild).mp. or school child.ti,ab. or school child*.ti,ab. or (adolescen* or juvenil* or youth* or teen* or under*age* or pubescen*).mp. or exp pediatrics/ or (pediatric* or paediatric* or peadiatric*).mp. or school.ti,ab. or school*.ti,ab. or (prematur* or preterm*).mp.) |
| 67. limit 66 to ("in data review" or in process or "pubmed not medline") |
| 68. 65 or 67 |

Embase

| 1. exp Vitamin D/ |
| --- |
| 2. (vitamin adj (d or d2 or d3)).tw. |
| 3. calcidiol.tw. |
| 4. ergocalciferol$.tw. |
| 5. cholecalciferol$.tw. |
| 6. calciferol.tw. |
| 7. Vitamin D Deficiency/dm, dt, th |
| 8. or/1-7 |
| 9. (25-hydroxyvitamin D or 25-hydroxy vitamin d or Plasma vitamin D).tw. |
| 10. 64719-49-9.rn. |
| 11. 25OHD3.tw. |
| 12. "25(OH)D3".tw. |
| 13. 25-OHD3.tw. |
| 14. "25-(OH)D3".tw. |
| 15. 25OHD.tw. |
| 16. "25(OH)D".tw. |
| 17. 25-OHD.tw. |
| 18. "25-(OH)D".tw. |
| 19. (25-hydroxycholecalciferol or 25-hydroxyergocalciferol).tw. |
| 20. plasma calcidiol.tw. |
| 21. (Urine calcium or (calcium adj3 ratio)).tw. |
| 22. or/9-21 |
| 23. exp Vitamin D Deficiency/ not Vitamin D Deficiency/dm, dt, th |
| 24. (avitaminosis and (d or d2 or d3)).tw. |
| 25. Vitamin D/to |
| 26. upper limit$.tw. |
| 27. UL.tw. |
| 28. (excess$ or toxic$).tw. |
| 29. (noael or noel).tw. |
| 30. (no observed adj2 effect$).tw. |
| 31. exp Calcification/ |
| 32. Hypercalcemia/ |
| 33. exp Stone Formation/ |
| 34. Calcinosis/ |
| 35. Hypercalcemi$.tw. |
| 36. (Burnett$ adj2 syndrome$).tw. |
| 37. Hypercalciuri$.tw. |
| 38. exp Vitamin d/ae |
| 39. (Side effect* or adverse effect$).tw. |
| 40. or/23-39 |
| 41. 8 and (22 or 40) |
| 42. ((single adj2 dose) or bolus or stoss* or single day or mega*).tw. |
| 43. Dose-Response Relationship, Drug/ |
| 44. 42 or 43 |
| 45. Randomized Controlled Trial/ or Single-blind Procedure/ or Crossover Procedure/ or Double-blind Procedure/ or random$.tw. or factorial$.tw. or crossover$.tw. or cross over$.tw. or cross-over$.tw. or placebo$.tw. or (doubl$ adj blind$).tw. or (singl$ adj blind$).tw. or assign$.tw. or allocat$.tw. or volunteer$.tw. |
| 46. 41 and 45 |
| 47. limit 41 to (clinical trial or randomized controlled trial or controlled clinical trial or multicenter study or phase 1 clinical trial or phase 2 clinical trial or phase 3 clinical trial or phase 4 clinical trial) |
| 48. 46 or 47 or 44 |
| 49. 8 and 48 |
| 50. limit 49 to (infant or child or preschool child <1 to 6 years> or school child <7 to 12 years> or adolescent <13 to 17 years>) |
| 51. 49 and ((Infan* or newborn* or new-born* or perinat* or neonat* or baby or baby* or babies or toddler* or minors or minors* or boy or boys or boyfriend or boyhood or girl* or kid or kids or child or child* or children* or schoolchild* or schoolchild).mp. or school child.ti,ab. or school child*.ti,ab. or (adolescen* or juvenil* or youth* or teen* or under*age* or pubescen*).mp. or exp pediatrics/ or (pediatric* or paediatric* or peadiatric*).mp. or school.ti,ab. or school*.ti,ab. or (prematur* or preterm*).mp.) |
| 52. 50 or 51 |
| 53. animals/ not humans/ |
| 54. 52 not 53 |
|  |
| **Cochrane Central Register of Controlled Trials** |
| 1. exp Vitamin d/ |
| 2. (vitamin adj (d or d2 or d3)).tw. |
| 3. Calcifediol/ |
| 4. calcidiol.tw. |
| 5. Ergocalciferols/ |
| 6. Ergocalciferol$.tw. |
| 7. Cholecalciferol/ |
| 8. Cholecalciferol$.tw. |
| 9. calciferol.tw. |
| 10. Vitamin D Deficiency/dh, dt |
| 11. or/1-10 |
| 12. (25-hydroxyvitamin D or 25-hydroxy vitamin d or Plasma vitamin D).tw. |
| 13. 25OHD3.tw. |
| 14. "25(OH)D3".tw. |
| 15. 25-OHD3.tw. |
| 16. "25-(OH)D3".tw. |
| 17. 25OHD.tw. |
| 18. "25(OH)D".tw. |
| 19. 25-OHD.tw. |
| 20. "25-(OH)D".tw. |
| 21. (25-hydroxycholecalciferol or 25-hydroxyergocalciferol).tw. |
| 22. Calcium/bl, ur |
| 23. plasma calcidiol.tw. |
| 24. (Urine calcium or (calcium adj3 ratio)).tw. |
| 25. exp Vitamin D Deficiency/ not Vitamin D Deficiency/dh, dt |
| 26. (avitaminosis and (d or d2 or d3)).tw. |
| 27. Vitamin D/to |
| 28. No-Observed-Adverse-Effect Level/ |
| 29. upper limit$.tw. |
| 30. UL.tw. |
| 31. (excess$ or toxic$).tw. |
| 32. (noael or noel).tw. |
| 33. (no observed adj2 effect$).tw. |
| 34. Calcification, Physiologic/de |
| 35. Hypercalcemia/ |
| 36. Kidney Calculi/ |
| 37. Nephrocalcinosis/ |
| 38. Urinary Calculi/ |
| 39. Bladder Calculi/ |
| 40. Ureteral Calculi/ |
| 41. Calcinosis/ |
| 42. Hypercalcemi$.tw. |
| 43. (Burnett$ adj2 syndrome$).tw. |
| 44. Hypercalciuri$.tw. |
| 45. exp Vitamin d/ae or Calcifediol/ae or Ergocalciferols/ae or Cholecalciferol/ae |
| 46. (Side effect* or adverse effect$).tw. |
| 47. ((single adj2 dose) or bolus or stoss* or single day or mega*).tw. |
| 48. Dose-Response Relationship, Drug/ |
| 49. exp Vitamin D/ |
| 50. (vitamin adj (d or d2 or d3)).tw. |
| 51. calcidiol.tw. |
| 52. ergocalciferol$.tw. |
| 53. cholecalciferol$.tw. |
| 54. calciferol.tw. |
| 55. or/49-54 |
| 56. (25-hydroxyvitamin D or 25-hydroxy vitamin d or Plasma vitamin D).tw. |
| 57. 25OHD3.tw. |
| 58. "25(OH)D3".tw. |
| 59. 25-OHD3.tw. |
| 60. "25-(OH)D3".tw. |
| 61. 25OHD.tw. |
| 62. "25(OH)D".tw. |
| 63. 25-OHD.tw. |
| 64. "25-(OH)D".tw. |
| 65. (25-hydroxycholecalciferol or 25-hydroxyergocalciferol).tw. |
| 66. plasma calcidiol.tw. |
| 67. (Urine calcium or (calcium adj3 ratio)).tw. |
| 68. exp Vitamin D Deficiency/ not Vitamin D Deficiency/dm, dt, th |
| 69. (avitaminosis and (d or d2 or d3)).tw. |
| 70. Vitamin D/to |
| 71. upper limit$.tw. |
| 72. UL.tw. |
| 73. (excess$ or toxic$).tw. |
| 74. (noael or noel).tw. |
| 75. (no observed adj2 effect$).tw. |
| 76. exp Calcification/ |
| 77. Hypercalcemia/ |
| 78. exp Stone Formation/ |
| 79. Calcinosis/ |
| 80. Hypercalcemi$.tw. |
| 81. (Burnett$ adj2 syndrome$).tw. |
| 82. Hypercalciuri$.tw. |
| 83. exp Vitamin d/ae |
| 84. (Side effect* or adverse effect$).tw. |
| 85. ((single adj2 dose) or bolus or stoss* or single day or mega*).tw. |
| 86. Dose-Response Relationship, Drug/ |
| 87. 85 or 86 |
| 88. 11 and (or/12-48) |
| 89. 55 and (or/56-86) |
| 90. 88 or 89 |
| 91. 90 and ((Infan* or newborn* or new-born* or perinat* or neonat* or baby or baby* or babies or toddler* or minors or minors* or boy or boys or boyfriend or boyhood or girl* or kid or kids or child or child* or children* or schoolchild* or schoolchild).mp. or school child.ti,ab. or school child*.ti,ab. or (adolescen* or juvenil* or youth* or teen* or under*age* or pubescen*).mp. or exp pediatrics/ or (pediatric* or paediatric* or peadiatric*).mp. or school.ti,ab. or school*.ti,ab. or (prematur* or preterm*).mp.) |
