# Supplementary material for: A systematic review of pediatric clinical trials of high dose vitamin D
Source: PeerJ. 2016 Feb 25;4:e1701. doi: 10.7717/peerj.1701 (PMC4782742; doi:10.7717/peerj.1701)
Supplement: Appendix S2 [file peerj-04-1701-s012.doc]

Bacchetta J, Ranchin B, Dubourg L, Cochat P 2010. [Vitamin D revisited: a cornerstone of health?]. *Archives de pédiatrie : organe officiel de la Sociéte française de pédiatrie* 17:1687–1695.

Barengolts E 2010. Vitamin D role and use in prediabetes. *Endocrine practice : official journal of the American College of Endocrinology and the American Association of Clinical Endocrinologists* 16:476–485.

Benjamin DK, Smith PB, Sun MJM, Murphy MD, Avant D, Mathis L, Rodriguez W, Califf RM, Li JS 2009. Safety and transparency of pediatric drug trials. *Archives of pediatrics & adolescent medicine* 163:1080–1086.

Cashman KD, Fitzgerald AP, Kiely M, Seamans KM 2011. A systematic review and meta-regression analysis of the vitamin D intake-serum 25-hydroxyvitamin D relationship to inform European recommendations. *British Journal of Nutrition* 106:1638–1648.

Chung M, Balk EM, Brendel M, Ip S, Lau J, Lee J, Lichtenstein A, Patel K, Raman G, Tatsioni A, Terasawa T, Trikalinos TA 2009. Vitamin D and calcium: a systematic review of health outcomes. *Evidence report/technology assessment*:1–420.

Cranney A, Horsley T, O'Donnell S, Weiler H, Puil L, Ooi D, Atkinson S, Ward L, Moher D, Hanley D, Fang M, Yazdi F, Garritty C, Sampson M, Barrowman N, Tsertsvadze A, Mamaladze V 2007. Effectiveness and safety of vitamin D in relation to bone health. *Evidence report/technology assessment*:1–235.

Cranney A, Weiler HA, O'Donnell S, Puil L 2008. Summary of evidence-based review on vitamin D efficacy and safety in relation to bone health. *American Journal of Clinical Nutrition* 88:513S–519S.

de Jager MEA, de Jong EMGJ, van de Kerkhof PCM, Seyger MMB 2010. Efficacy and safety of treatments for childhood psoriasis: a systematic literature review. *Journal of the American Academy of Dermatology* 62:1013–1030.

Dolinsky DH, Armstrong S, Mangarelli C, Kemper AR 2013. The association between vitamin D and cardiometabolic risk factors in children: a systematic review. *Clinical pediatrics* 52:210–223.

Ferguson JH, Chang AB 2012. Vitamin D supplementation for cystic fibrosis. *Cochrane Database of Systematic Reviews* 4:CD007298.

Geary DF, Hodson EM, Craig JC 2010. Interventions for bone disease in children with chronic kidney disease. *Cochrane Database of Systematic Reviews*:CD008327.

Grossmann RE, Tangpricha V 2010. Evaluation of vehicle substances on vitamin D bioavailability: a systematic review. *Molecular nutrition & food research* 54:1055–1061.

Irlam JH, Visser MM, Rollins NN, Siegfried N 2010. Micronutrient supplementation in children and adults with HIV infection. *Cochrane Database of Systematic Reviews*:CD003650.

Lerch C, Meissner T 2007. Interventions for the prevention of nutritional rickets in term born children. *Cochrane Database of Systematic Reviews*:CD006164.

Nisar MK, Masood F, Cookson P, Sansome A, Ostör AJK 2013. What do we know about juvenile idiopathic arthritis and vitamin D? A systematic literature review and meta-analysis of current evidence. *Clinical rheumatology* 32:729–734.

O'Donnell S, Cranney A, Horsley T, Weiler HA, Atkinson SA, Hanley DA, Ooi DS, Ward L, Barrowman N, Fang M, Sampson M, Tsertsvadze A, Yazdi F 2008. Efficacy of food fortification on serum 25-hydroxyvitamin D concentrations: systematic review. *American Journal of Clinical Nutrition* 88:1528–1534.

Onwuneme C, Carroll A, McCarthy R, Kilbane M, McKenna M, Murphy N, Molloy EJ 2012. Towards evidence based medicine for paediatricians. Question 2. What is the ideal dose of vitamin D supplementation for term neonates? *Archives of Disease in Childhood* 97:387–389.

Palmer SC, McGregor DO, Craig JC, Elder G, Macaskill P, Strippoli GF 2009. Vitamin D compounds for people with chronic kidney disease not requiring dialysis. *Cochrane Database of Systematic Reviews*:CD008175.

Seamans KM, Cashman KD 2009. Existing and potentially novel functional markers of vitamin D status: a systematic review. *American Journal of Clinical Nutrition* 89:1997S–2008S.

Tangpricha V, Kelly A, Stephenson A, Maguiness K, Enders J, Robinson KA, Marshall BC, Borowitz D, Cystic Fibrosis Foundation Vitamin D Evidence-Based Review Committee 2012. An update on the screening, diagnosis, management, and treatment of vitamin D deficiency in individuals with cystic fibrosis: evidence-based recommendations from the Cystic Fibrosis Foundation. *Journal of Clinical Endocrinology & Metabolism* 97:1082–1093.

Thornton J, Ashcroft D, O'Neill T, Elliott R, Adams J, Roberts C, Rooney M, Symmons D 2008. A systematic review of the effectiveness of strategies for reducing fracture risk in children with juvenile idiopathic arthritis with additional data on long-term risk of fracture and cost of disease management. *Health technology assessment (Winchester, England)* 12:iii–ix– xi–xiv– 1–208.

Winzenberg TM, Powell S, Shaw KA, Jones G 2010. Vitamin D supplementation for improving bone mineral density in children. *Cochrane Database of Systematic Reviews*:CD006944.

Winzenberg T, Powell S, Shaw KA, Jones G 2011. Effects of vitamin D supplementation on bone density in healthy children: systematic review and meta-analysis. *BMJ* 342:c7254.

Zipitis CS, Akobeng AK 2008. Vitamin D supplementation in early childhood and risk of type 1 diabetes: a systematic review and meta-analysis. *Archives of Disease in Childhood* 93:512–517.
