# Supplementary material for: A systematic review of pediatric clinical trials of high dose vitamin D
Source: PeerJ. 2016 Feb 25;4:e1701. doi: 10.7717/peerj.1701 (PMC4782742; doi:10.7717/peerj.1701)
Supplement: Appendix S3 [file peerj-04-1701-s013.docx]

**1.** Zuckerman M, Pettifor JM. Rickets in very-low-birth-weight infants born at Baragwanath Hospital. *S. Afr. Med. J.* Vol 841994:216-220.

**2.** Zeghoud F, Vervel C, Guillozo H, Walrant-Debray O, Boutignon H, Garabedian M. Subclinical vitamin D deficiency in neonates: definition and response to vitamin D supplements. *Am J Clin Nutr.* Vol 651997:771-778.

**3.** Zeghoud F, Delaveyne R, Rehel P, Chalas J, Garabedian M, Odievre M. [Vitamin D and pubertal maturation. Value and tolerance of vitamin D supplementation during the winter season]. *Arch Pediatr.* Vol 21995:221-226.

**4.** Zeghoud F, Ben-Mekhbi H, Djeghri N, Garabedian M. Vitamin D prophylaxis during infancy: comparison of the long-term effects of three intermittent doses (15, 5, or 2.5 mg) on 25-hydroxyvitamin D concentrations. *Am J Clin Nutr.* Vol 601994:393-396.

**5.** Wicklow BA, Taback SP. Feasibility of a type 1 diabetes primary prevention trial using 2000 IU vitamin D3 in infants from the general population with increased HLA-associated risk. *Ann. N. Y. Acad. Sci.* Vol 10792006:310-312.

**6.** Westfechtel A, Tuschen T, Otten A, Wolf H. [Hypophosphatemic rickets in premature infants weighing less than 1500 grams on oral and parenteral feeding]. *Monatsschr Kinderheilkd.* Vol 1321984:212-216.

**7.** Wagner E, Czermak H, Swoboda W. [Effect of oral and intramuscular massive vitamin D doses on serum calcium in premature infants]. *Wien Med Wochenschr.* Vol 1191969:415-418.

**8.** Vervel C, Zeghoud F, Boutignon H, Tjani JC, Walrant-Debray O, Garabedian M. [Fortified milk and supplements of oral vitamin D. Comparison of the effect of two doses of vitamin D (500 and 1,000 UI/d) during the first trimester of life]. *Arch Pediatr.* Vol 41997:126-132.

**9.** Tiosano D, Weisman Y, Hochberg Z. The role of the vitamin D receptor in regulating vitamin D metabolism: a study of vitamin D-dependent rickets, type II. *J Clin Endocrinol Metab.* Vol 862001:1908-1912.

**10.** Thacher TD, Obadofin MO, O’Brien KO, Abrams SA. The effect of vitamin D2 and vitamin D3 on intestinal calcium absorption in Nigerian children with rickets. *J Clin Endocrinol Metab.* Vol 942009:3314-3321.

**11.** Thacher TD, Fischer PR, Pettifor JM, Lawson JO, Isichei CO, Reading JC, Chan GM. A comparison of calcium, vitamin D, or both for nutritional rickets in Nigerian children. *N. Engl. J. Med.* Vol 3411999:563-568.

**12.** Thacher TD, Fischer PR, Isichei CO, Pettifor JM. Early response to vitamin D2 in children with calcium deficiency rickets. *J Pediatr.* Vol 1492006:840-844.

**13.** Thacher TD, Aliu O, Griffin IJ, Pam SD, O’Brien KO, Imade GE, Abrams SA. Meals and dephytinization affect calcium and zinc absorption in Nigerian children with rickets. *J Nutr.* Vol 1392009:926-932.

**14.** Tau C, Garabedian M, Farriaux JP, Czernichow P, Pomarede R, Balsan S. Hypercalcemia in infants with congenital hypothyroidism and its relation to vitamin D and thyroid hormones. *J Pediatr.* Vol 1091986:808-814.

**15.** Tau C, Ciriani V, Scaiola E, Acuña M. Twice single doses of 100,000 IU of vitamin D in winter is adequate and safe for prevention of vitamin D deficiency in healthy children from Ushuaia, Tierra Del Fuego, Argentina. *J Steroid Biochem Mol Biol.* Vol 1032007:651-654.

**16.** Stogmann W, Sacher M, Blümel P, Woloszczuk W. [Vitamin D deficiency rickets: single-dose therapy versus continuous therapy]. *Padiatr Padol.* Vol 201985:385-392.

**17.** Soliman AT, El-Dabbagh M, Adel A, Al Ali M, Aziz Bedair EM, Elalaily RK. Clinical responses to a mega-dose of vitamin D3 in infants and toddlers with vitamin D deficiency rickets. *J Trop Pediatr.* Vol 562010:19-26.

**18.** Soliman AT, Al Khalaf F, Alhemaidi N, Al Ali M, Al Zyoud M, Yakoot K. Linear growth in relation to the circulating concentrations of insulin-like growth factor I, parathyroid hormone, and 25-hydroxy vitamin D in children with nutritional rickets before and after treatment: endocrine adaptation to vitamin D deficiency. *Metab. Clin. Exp.* Vol 572008:95-102.

**19.** Soliman A, Adel A, Wagdy M, Al Ali M, ElMulla N. Calcium homeostasis in 40 adolescents with beta-thalassemia major: a case-control study of the effects of intramuscular injection of a megadose of cholecalciferol. *Pediatr Endocrinol Rev.* Vol 6 Suppl 12008:149-154.

**20.** Silver J, Davies TJ, Kupersmitt E, Orme M, Petrie A, Vajda F. Prevalence and treatment of vitamin D deficiency in children on anticonvulsant drugs. *Arch Dis Child.* Vol 491974:344-350.

**21.** Sidbury R, Sullivan AF, Thadhani RI, Camargo CA. Randomized controlled trial of vitamin D supplementation for winter-related atopic dermatitis in Boston: a pilot study. *Br. J. Dermatol.* Vol 1592008:245-247.

**22.** Shakiba M, Sadr S, Nefei Z, Mozaffari-Khosravi H, Lotfi MH, Bemanian MH. Combination of bolus dose vitamin D with routine vaccination in infants: a randomised trial. *Singapore Med J.* Vol 512010:440-445.

**23.** Shajari A, Shakiba M, Nourani F, Zaki M, Kheirandish M. Urinary Calcium/Creatinin Ratio with Different Dosages of Vitamin D3 Prophylaxis in Infants. *Iranian Journal of Pediatrics.* Vol 192009:159-163.

**24.** Schmidt P, Szilagyi I, Ecsi M, Varga R. [Preliminary experiences in the prevention of rickets with reduced vitamin D doses]. *Kinderarztl Prax.* Vol 561988:179-182.

**25.** Robinson MJ, Merrett AL, Tetlow VA, Compston JE. Plasma 25-hydroxyvitamin D concentrations in preterm infants receiving oral vitamin D supplements. *Arch Dis Child.* Vol 561981:144-145.

**26.** Reinken L, Obladen M, Dockx-Reinken F, Lindemann C. [The effect of osteopenia prevention in very small premature infants on hormonal parameters of calcium metabolism and bone mineralization]. *Klin Padiatr.* Vol 2011989:177-182.

**27.** Raghuramulu N, Reddy V. Studies on vitamin D metabolism in malnourished children. *Br J Nutr.* Vol 471982:231-234.

**28.** Radde IC, Chance GW, Bailey K, O’Brien J, Day GM, Sheepers J. Growth and mineral metabolism in very low birth weight infants. I. Comparison of the effects of two modes of NaHCO3 treatment of late metabolic acidosis. *Pediatr Res.* Vol 91975:564-568.

**29.** Pittard WB, Geddes KM, Hulsey TC, Hollis BW. How much vitamin D for neonates? *Am. J. Dis. Child.* Vol 1451991:1147-1149.

**30.** Pettifor JM, Stein H, Herman A, Ross FP, Blumenfeld T, Moodley GP. Mineral homeostasis in very low birth weight infants fed either own mother’s milk or pooled pasteurized preterm milk. *J Pediatr Gastroenterol Nutr.* Vol 51986:248-253.

**31.** Pettifor JM, Rajah R, Venter A, Moodley GP, Opperman L, Cavaleros M, Ross FP. Bone mineralization and mineral homeostasis in very low-birth-weight infants fed either human milk or fortified human milk. *J Pediatr Gastroenterol Nutr.* Vol 81989:217-224.

**32.** Pehlivan I, Hatun S, Aydoğan M, Babaoğlu K, Gökalp AS. Maternal vitamin D deficiency and vitamin D supplementation in healthy infants. *Turk. J. Pediatr.* Vol 452003:315-320.

**33.** Oliveri B, Cassinelli H, Mautalen C, Ayala M. Vitamin D prophylaxis in children with a single dose of 150000 IU of vitamin D. *Eur J Clin Nutr.* Vol 501996:807-810.

**34.** Moya M, Beltran J, Colomer J. Therapeutic and collateral effects of 25-hydroxycholecalciferol in vitamin D deficiency. *Eur J Pediatr.* Vol 1271977:49-55.

**35.** Morcos MM, Gabr AA, Samuel S, Kamel M, el Baz M, el Beshry M, Michail RR. Vitamin D administration to tuberculous children and its value. *Boll Chim Farm.* Vol 1371998:157-164.

**36.** Mikati MA, Dib L, Yamout B, Sawaya R, Rahi AC, Fuleihan GE-H. Two randomized vitamin D trials in ambulatory patients on anticonvulsants: impact on bone. *Neurology.* Vol 672006:2005-2014.

**37.** Mawer EB, Stanbury W, Robinson MJ, James J, Close C. Vitamin D nutrition and vitamin D metabolism in the premature human neonate. *Clin. Endocrinol. (Oxf).* Vol 251986:641-649.

**38.** Masood H, Narang AP, Bhat IA, Shah GN. Persistent limb pain and raised serum alkaline phosphatase the earliest markers of subclinical hypovitaminosis D in Kashmir. *Indian J. Physiol. Pharmacol.* Vol 331989:259-261.

**39.** Markestad T, Hesse V, Siebenhuner M, Jahreis G, Aksnes L, Plenert W, Aarskog D. Intermittent high-dose vitamin D prophylaxis during infancy: effect on vitamin D metabolites, calcium, and phosphorus. *Am J Clin Nutr.* Vol 461987:652-658.

**40.** Markestad T, Halvorsen S, Halvorsen KS, Aksnes L, Aarskog D. Plasma concentrations of vitamin D metabolites before and during treatment of vitamin D deficiency rickets in children. *Acta Paediatr Scand.* Vol 731984:225-231.

**41.** Majak P, Rychlik B, Stelmach I. The effect of oral steroids with and without vitamin D3 on early efficacy of immunotherapy in asthmatic children. *Clin Exp Allergy.* Vol 392009:1830-1841.

**42.** Maalouf J, Nabulsi M, Vieth R, Kimball S, El-Rassi R, Mahfoud Z, El-Hajj Fuleihan G. Short- and long-term safety of weekly high-dose vitamin D3 supplementation in school children. *J Clin Endocrinol Metab.* Vol 932008:2693-2701.

**43.** Lubani MM, al-Shab TS, al-Saleh QA, Sharda DC, Quattawi SA, Ahmed SA, Moussa MA, Reavey PC. Vitamin-D-deficiency rickets in Kuwait: the prevalence of a preventable disease. *Ann Trop Paediatr.* Vol 91989:134-139.

**44.** Liakakos D, Papadopoulos Z, Vlachos P, Boviatsi E, Varonos DD. Serum alkaline phosphatase and urinary hydroxyproline values in children receiving phenobarbital with and without vitamin D. *J Pediatr.* Vol 871975:291-296.

**45.** Lehtonen-Veromaa M, Mottonen T, Nuotio I, Irjala K, Viikari J. The effect of conventional vitamin D(2) supplementation on serum 25(OH)D concentration is weak among peripubertal Finnish girls: a 3-y prospective study. *Eur J Clin Nutr.* Vol 562002:431-437.

**46.** Leger J, Tau C, Garabedian M, Farriaux JP, Czernichow P. [Prophylaxis of vitamin D deficiency in hypothyroidism in the newborn infant]. *Arch. Fr. Pediatr.* Vol 461989:567-571.

**47.** Kutluk G, Cetinkaya F, Başak M. Comparisons of oral calcium, high dose vitamin D and a combination of these in the treatment of nutritional rickets in children. *J Trop Pediatr.* Vol 482002:351-353.

**48.** Kunz C, von Lilienfeld-Toal H, Niesen M, Burmeister W. [25-hydroxy-vitamin-D in serum of newborns and infants during continuous oral vitamin D treatment (author’s transl)]. *Padiatr Padol.* Vol 171982:181-185.

**49.** Kislal FM, Dilmen U. Effect of different doses of vitamin D on osteocalcin and deoxypyridinoline in preterm infants. *Pediatr Int.* Vol 502008:204-207.

**50.** Kilpinen-Loisa P, Nenonen H, Pihko H, Mäkitie O. High-dose vitamin D supplementation in children with cerebral palsy or neuromuscular disorder. *Neuropediatrics.* Vol 382007:167-172.

**51.** Khadilkar AV, Sayyad MG, Sanwalka NJ, Bhandari DR, Naik S, Khadilkar VV, Mughal MZ. Vitamin D supplementation and bone mass accrual in underprivileged adolescent Indian girls. *Asia Pac J Clin Nutr.* Vol 192010:465-472.

**52.** Hoppe B, Hesse A, Neuhaus T, Fanconi S, Blau N, Roth B, Leumann E. Influence of nutrition on urinary oxalate and calcium in preterm and term infants. *Pediatr Nephrol.* Vol 111997:687-690.

**53.** Holst-Gemeiner D, Gemeiner M, Pilz I, Swoboda W. [Plasma 25-hydroxycholecalciferol after daily vitamin D administration in comparison with massive single-dose prophylaxis (author’s transl)]. *Wien. Klin. Wochenschr.* Vol 901978:509-512.

**54.** Hodson EM, Evans RA, Dunstan CR, Hills E, Wong SY, Rosenberg AR, Roy LP. Treatment of childhood renal osteodystrophy with calcitriol or ergocalciferol. *Clin. Nephrol.* Vol 241985:192-200.

**55.** Hillman LS, Hollis B, Salmons S, Martin L, Slatopolsky E, McAlister W, Haddad J. Absorption, dosage, and effect on mineral homeostasis of 25-hydroxycholecalciferol in premature infants: comparison with 400 and 800 IU vitamin D2 supplementation. *J Pediatr.* Vol 1061985:981-989.

**56.** Hillman LS, Cassidy JT, Popescu MF, Hewett JE, Kyger J, Robertson JD. Percent true calcium absorption, mineral metabolism, and bone mineralization in children with cystic fibrosis: effect of supplementation with vitamin D and calcium. *Pediatr. Pulmonol.* Vol 432008:772-780.

**57.** Hillman LS, Cassidy JT, Chanetsa F, Hewett JE, Higgins BJ, Robertson JD. Percent true calcium absorption, mineral metabolism, and bone mass in children with arthritis: effect of supplementation with vitamin D3 and calcium. *Arthritis Rheum.* Vol 582008:3255-3263.

**58.** Heubi JE, Hollis BW, Tsang RC. Bone disease in chronic childhood cholestasis. II. Better absorption of 25-OH vitamin D than vitamin D in extrahepatic biliary atresia. *Pediatr Res.* Vol 271990:26-31.

**59.** Heubi JE, Hollis BW, Specker B, Tsang RC. Bone disease in chronic childhood cholestasis. I. Vitamin D absorption and metabolism. *Hepatology.* Vol 91989:258-264.

**60.** Henriksen C, Helland IB, Ronnestad A, Gronn M, Iversen PO, Drevon CA. Fat-soluble vitamins in breast-fed preterm and term infants. *Eur J Clin Nutr.* Vol 602006:756-762.

**61.** Hawker GA, Ridout R, Harris VA, Chase CC, Fielding LJ, Biggar WD. Alendronate in the treatment of low bone mass in steroid-treated boys with Duchennes muscular dystrophy. *Arch Phys Med Rehabil.* Vol 862005:284-288.

**62.** Gupta SK, Gupta RC, Seth AK, Gupta A. Reversal of fluorosis in children. *Acta Paediatr Jpn.* Vol 381996:513-519.

**63.** Gupta SK, Gupta RC, Seth AK. Reversal of clinical and dental fluorosis. *Indian Pediatr.* Vol 311994:439-443.

**64.** Guillemant J, Le HT, Maria A, Allemandou A, Pérès G, Guillemant S. Wintertime vitamin D deficiency in male adolescents: effect on parathyroid function and response to vitamin D3 supplements. *Osteoporos.Int.* Vol 122001:875-879.

**65.** Guillemant J, Allemandou A, Cabrol S, Pérès G, Guillemant S. [Vitamin D status in the adolescent: seasonal variations and effects of winter supplementation with vitamin D3]. *Arch Pediatr.* Vol 51998:1211-1215.

**66.** Gordon CM, Williams AL, Feldman HA, May J, Sinclair L, Vasquez A, Cox JE. Treatment of hypovitaminosis D in infants and toddlers. *J Clin Endocrinol Metab.* Vol 932008:2716-2721.

**67.** Goncerzewicz M, Ryzko J, Lorenc R, Kozlowski K, Socha J. [Vitamin D metabolism in children with malabsorption syndrome]. *Klin Padiatr.* Vol 1971985:30-34.

**68.** Glorieux FH, Salle BL, Delvin EE, David L. Vitamin D metabolism in preterm infants: serum calcitriol values during the first five days of life. *J Pediatr.* Vol 991981:640-643.

**69.** Effect of single massive dose of vitamin D therapy (oral or intramuscular) on rickets in Addis Ababa children [computer program]. 1972: Courrier.

**70.** Forin V, Arabi A, Guigonis V, Filipe G, Bensman A, Roux C. Benefits of pamidronate in children with osteogenesis imperfecta: an open prospective study. *Joint Bone Spine.* Vol 722005:313-318.

**71.** Evans JR, Allen AC, Stinson DA, Hamilton DC, St John Brown B, Vincer MJ, Raad MA, Gundberg CM, Cole DE. Effect of high-dose vitamin D supplementation on radiographically detectable bone disease of very low birth weight infants. *J Pediatr.* Vol 1151989:779-786.

**72.** El-Hajj Fuleihan G, Nabulsi M, Tamim H, Maalouf J, Salamoun M, Khalife H, Choucair M, Arabi A, Vieth R. Effect of vitamin D replacement on musculoskeletal parameters in school children: a randomized controlled trial. *J Clin Endocrinol Metab.* Vol 912006:405-412.

**73.** Eke FU, Winterborn MH. Effect of low dose 1 alpha-hydroxycholecalciferol on glomerular filtration rate in moderate renal failure. *Arch Dis Child.* Vol 581983:810-813.

**74.** Edda LF, Pilar AG, Pablo VC, Felipe HR, Marisol CA, Arnaldo CJPMaFC. Effects of two forms of vitamin D supplementation on bone metabolism and infants growth. *Revista Chilena de Pediatria.* Vol 671996:219-223.

**75.** Duhamel JF, Zeghoud F, Sempe M, Boudailliez B, Odievre M, Laurans M, Garabedian M, Mallet E. [Prevention of vitamin D deficiency in adolescents and pre-adolescents. An interventional multicenter study on the biological effect of repeated doses of 100,000 IU of vitamin D3]. *Arch Pediatr.* Vol 72000:148-153.

**76.** Delvin EE, Salle BL, Claris O, Putet G, Hascoet J-M, Desnoulez L, Messai S, Lévy E. Oral vitamin A, E and D supplementation of pre-term newborns either breast-fed or formula-fed: a 3-month longitudinal study. *J Pediatr Gastroenterol Nutr.* Vol 402005:43-47.

**77.** Day GM, Chance GW, Radde IC, Reilly BJ, Park E, Sheepers J. Growth and mineral metabolism in very low birth weight infants. II. Effects of calcium supplementation on growth and divalent cations. *Pediatr Res.* Vol 91975:568-575.

**78.** Dahifar H, Faraji A, Yassobi S, Ghorbani A. Asymptomatic rickets in adolescent girls. *Indian J.Pediatr.* Vol 742007:571-575.

**79.** Dahifar H, Faraji A, Ghorbani A, Yassobi S. Impact of dietary and lifestyle on vitamin D in healthy student girls aged 11-15 years. *J. Med. Invest.* Vol 532006:204-208.

**80.** Christiansen C, Røodbro P, Sjö. Biochemical status in epileptic patients during treatment with vitamin D. A controlled therapeutic trial. *Acta Neurol. Scand.* Vol 521975:81-86.

**81.** Christiansen C, Rødbro P, Nielsen CT. Iatrogenic osteomalacia in epileptic children. A controlled therapeutic trial. *Acta Paediatr Scand.* Vol 641975:219-224.

**82.** Cesur Y, Caksen H, Gündem A, Kirimi E, Odabaş D. Comparison of low and high dose of vitamin D treatment in nutritional vitamin D deficiency rickets. *J. Pediatr. Endocrinol. Metab.* Vol 162003:1105-1109.

**83.** Boas SR, Hageman JR, Ho LT, Liveris M. Very high-dose ergocalciferol is effective for correcting vitamin D deficiency in children and young adults with cystic fibrosis. *J Cyst.Fibros.* Vol 82009:270-272.

**84.** Billoo AG, Murtaza G, Memon MA, Khaskheli SA, Iqbal K, Rao MH. Comparison of oral versus injectable vitamin-D for the treatment of nutritional vitamin-D deficiency rickets. *J Coll Physicians Surg Pak.* Vol 192009:428-431.

**85.** Bereket A, Cesur Y, Özkan B, Adal E, Turan S, Onan SH, Döneray H, Akçay T, Haklar G. Circulating insulin-like growth factor binding protein-4 (IGFBP-4) is not regulated by parathyroid hormone and vitamin D in vivo: evidence from children with rickets. *J Clin Res Pediatr Endocrinol.* Vol 22010:17-20.

**86.** Baroncelli GI, Bertelloni S, Ceccarelli C, Amato V, Saggese G. Bone turnover in children with vitamin D deficiency rickets before and during treatment. *Acta Paediatr.* Vol 892000:513-518.

**87.** Balasubramanian K, Rajeswari J, Gulab, Govil YC, Agarwal AK, Kumar A, Bhatia V. Varying role of vitamin D deficiency in the etiology of rickets in young children vs. adolescents in northern India. *J Trop Pediatr.* Vol 492003:201-206.

**88.** Backstrom MC, Maki R, Kuusela AL, Sievanen H, Koivisto AM, Koskinen M, Ikonen RS, Mäki M. The long-term effect of early mineral, vitamin D, and breast milk intake on bone mineral status in 9- to 11-year-old children born prematurely. *J Pediatr Gastroenterol Nutr.* Vol 291999:575-582.

**89.** Backstrom MC, Maki R, Kuusela AL, Sievanen H, Koivisto AM, Ikonen RS, Kouri T, Mäki M. Randomised controlled trial of vitamin D supplementation on bone density and biochemical indices in preterm infants. *Arch. Dis. Child. Fetal Neonatal Ed.* Vol 801999:F161-166.

**90.** Arpadi SM, McMahon D, Abrams EJ, Bamji M, Purswani M, Engelson ES, Horlick M, Shane E. Effect of bimonthly supplementation with oral cholecalciferol on serum 25-hydroxyvitamin D concentrations in HIV-infected children and adolescents. *Pediatrics.* Vol 1232009:e121-126.

**91.** Argao EA, Heubi JE, Hollis BW, Tsang RC. d-Alpha-tocopheryl polyethylene glycol-1000 succinate enhances the absorption of vitamin D in chronic cholestatic liver disease of infancy and childhood. *Pediatr Res.* Vol 311992:146-150.

**92.** Arabi A, Zahed L, Mahfoud Z, El-Onsi L, Nabulsi M, Maalouf J, Fuleihan GE-H. Vitamin D receptor gene polymorphisms modulate the skeletal response to vitamin D supplementation in healthy girls. *Bone.* Vol 452009:1091-1097.

**93.** Andersen R, Mølgaard C, Skovgaard LT, Brot C, Cashman KD, Jakobsen J, Lamberg-Allardt C, Ovesen L. Effect of vitamin D supplementation on bone and vitamin D status among Pakistani immigrants in Denmark: a randomised double-blinded placebo-controlled intervention study. *Br J Nutr.* Vol 1002008:197-207.

**94.** Alizade P, Naderi F, Sotoudeh K. A randomized clinical trial of prophylactic effects of vitamin D on different indices of osteopenia of prematurity. *Iranian Journal of Public Health.* Vol 352006:58-63.

**95.** Ala-Houhala M. 25-Hydroxyvitamin D levels during breast-feeding with or without maternal or infantile supplementation of vitamin D. *J Pediatr Gastroenterol Nutr.* Vol 41985:220-226.

**96.** Akcam M, Yildiz M, Yilmaz A, Artan R. Bone mineral density in response to two different regimes in rickets. *Indian Pediatr.* Vol 432006:423-427.

**97.** Ziegler EE, Nelson SE, Jeter JM. Vitamin D supplementation of breastfed infants: a randomized dose-response trial. *Pediatr Res.* Vol 762014:177-183.

**98.** Yadav M, Mittal K. Effect of vitamin D supplementation on moderate to severe bronchial asthma. *Indian J.Pediatr.* Vol 812014:650-654.

**99.** Wingate KE, Jacobson K, Issenman R, Carroll M, Barker C, Israel D, Brill H, Weiler H, Barr SI, Li W, Lyon MR, Green TJ. 25-Hydroxyvitamin D concentrations in children with Crohn’s disease supplemented with either 2000 or 400 IU daily for 6 months: a randomized controlled study. *J Pediatr.* Vol 1642014:860-865.

**100.** Ward KA, Das G, Roberts SA, Berry JL, Adams JE, Rawer R, Mughal MZ. A randomized, controlled trial of vitamin D supplementation upon musculoskeletal health in postmenarchal females. *J Clin Endocrinol Metab.* Vol 952010:4643-4651.

**101.** Urashima M, Segawa T, Okazaki M, Kurihara M, Wada Y, Ida H. Randomized trial of vitamin D supplementation to prevent seasonal influenza A in schoolchildren. *Am J Clin Nutr.* Vol 912010:1255-1260.

**102.** Trilok-Kumar G, Arora H, Rajput M, Chellani H, Singh V, Raynes J, Arya S, Aggarwal S, Srivastava N, Sachdev HPS, Filteau S. Effect of vitamin D supplementation of low birth weight term Indian infants from birth on cytokine production at 6 months. *Eur J Clin Nutr.* Vol 662012:746-750.

**103.** Thacher TD, Fischer PR, Pettifor JM. Vitamin D treatment in calcium-deficiency rickets: a randomised controlled trial. *Arch Dis Child.* Vol 992014:807-811.

**104.** Thacher TD, Fischer PR, Obadofin MO, Levine MA, Singh RJ, Pettifor JM. Comparison of metabolism of vitamins D2 and D3 in children with nutritional rickets. *J Bone Miner.Res.* Vol 252010:1988-1995.

**105.** Tergestina M, Jose A, Sridhar S, Job V, Rebekah G, Kuruvilla KA, Thomas N. Vitamin D status and adequacy of standard supplementation in preterm neonates from South India. *J Pediatr Gastroenterol Nutr.* Vol 582014:661-665.

**106.** Soliman AT, Eldabbagh M, Elawwa A, Ashour R, Saleem W. The effect of vitamin D therapy on hematological indices in children with vitamin D deficiency. *J Trop Pediatr.* Vol 582012:523-524.

**107.** Soliman AT, Adel A, Wagdy M, Alali M, Aziz Bedair EM. Manifestations of severe vitamin D deficiency in adolescents: effects of intramuscular injection of a megadose of cholecalciferol. *J Trop Pediatr.* Vol 572011:303-306.

**108.** Soliman A, De Sanctis V, Adel A, El Awwa A, Bedair S. Clinical, biochemical and radiological manifestations of severe vitamin d deficiency in adolescents versus children: response to therapy. *Georgian Med News*2012:58-64.

**109.** Siafarikas A, Piazena H, Feister U, Bulsara MK, Meffert H, Hesse V. Randomised controlled trial analysing supplementation with 250 versus 500 units of vitamin D3, sun exposure and surrounding factors in breastfed infants. *Arch Dis Child.* Vol 962011:91-95.

**110.** Shroff R, Wan M, Gullett A, Ledermann S, Shute R, Knott C, Wells D, Aitkenhead H, Manickavasagar B, van’t Hoff W, Rees L. Ergocalciferol supplementation in children with CKD delays the onset of secondary hyperparathyroidism: a randomized trial. *Clin J Am Soc Nephrol.* Vol 72012:216-223.

**111.** Shneider BL, Magee JC, Bezerra JA, Haber B, Karpen SJ, Raghunathan T, Rosenthal P, Schwarz K, Suchy FJ, Kerkar N, Turmelle Y, Whitington PF, Robuck PR, Sokol RJ, (ChiLDREN) CLDREN. Efficacy of fat-soluble vitamin supplementation in infants with biliary atresia. *Pediatrics.* Vol 1302012:e607-614.

**112.** Shepherd D, Belessis Y, Katz T, Morton J, Field P, Jaffe A. Single high-dose oral vitamin D3 (stoss) therapy--a solution to vitamin D deficiency in children with cystic fibrosis? *J Cyst.Fibros.* Vol 122013:177-182.

**113.** Shedeed SA. Vitamin D supplementation in infants with chronic congestive heart failure. *Pediatr Cardiol.* Vol 332012:713-719.

**114.** Shakinba M, Tefagh S, Nafei Z. The optimal dose of vitamin D in growing girls during academic years: a randomized trial. *Turkish Journal of Medical Sciences.* Vol 412011:33-37.

**115.** Shakiba M, Pahloosye A, Mirouliaei M, Islami Z. Comparison of two regimens of vitamin D supplementation for vitamin D-deficient neonates. *Singapore Med J.* Vol 552014:266-270.

**116.** Shakiba M, Ghadir M, Nafei Z, Akhavan Karbasi S, Lotfi MH, Shajari A. Study to evaluate two dosage regimens of vitamin D through an academic year in middle school girls: a randomized trial. *Acta Med Iran.* Vol 492011:780-783.

**117.** Rich-Edwards JW, Ganmaa D, Kleinman K, Sumberzul N, Holick MF, Lkhagvasuren T, Dulguun B, Burke A, Frazier AL. Randomized trial of fortified milk and supplements to raise 25-hydroxyvitamin D concentrations in schoolchildren in Mongolia. *Am J Clin Nutr.* Vol 942011:578-584.

**118.** Rianthavorn P, Boonyapapong P. Ergocalciferol decreases erythropoietin resistance in children with chronic kidney disease stage 5. *Pediatr Nephrol.* Vol 282013:1261-1266.

**119.** Putman MS, Pitts SAB, Milliren CE, Feldman HA, Reinold K, Gordon CM. A randomized clinical trial of vitamin D supplementation in healthy adolescents. *J Adolesc Health.* Vol 522013:592-598.

**120.** Principi N, Marchisio P, Terranova L, Zampiero A, Baggi E, Daleno C, Tirelli S, Pelucchi C, Esposito S. Impact of vitamin D administration on immunogenicity of trivalent inactivated influenza vaccine in previously unvaccinated children. *Hum Vaccin Immunother.* Vol 92013:969-974.

**121.** Poomthavorn P, Nantarakchaikul P, Mahachoklertwattana P, Chailurkit L-o, Khlairit P. Effects of correction of vitamin D insufficiency on serum osteocalcin and glucose metabolism in obese children. *Clin. Endocrinol. (Oxf).* Vol 802014:516-523.

**122.** Park CY, Hill KM, Elble AE, Martin BR, DiMeglio LA, Peacock M, McCabe GP, Weaver CM. Daily supplementation with 25 μg cholecalciferol does not increase calcium absorption or skeletal retention in adolescent girls with low serum 25-hydroxyvitamin D. *J Nutr.* Vol 1402010:2139-2144.

**123.** Pappa HM, Mitchell PD, Jiang H, Kassiff S, Filip-Dhima R, DiFabio D, Quinn N, Lawton RC, Bronzwaer ME, Koenen M, Gordon CM. Maintenance of optimal vitamin D status in children and adolescents with inflammatory bowel disease: a randomized clinical trial comparing two regimens. *J Clin Endocrinol Metab.* Vol 992014:3408-3417.

**124.** Osunkwo I, Ziegler TR, Alvarez J, McCracken C, Cherry K, Osunkwo CE, Ofori-Acquah SF, Ghosh S, Ogunbobode A, Rhodes J, Eckman JR, Dampier C, Tangpricha V. High dose vitamin D therapy for chronic pain in children and adolescents with sickle cell disease: results of a randomized double blind pilot study. *Br. J. Haematol.* Vol 1592012:211-215.

**125.** Natarajan CK, Sankar MJ, Agarwal R, Pratap OT, Jain V, Gupta N, Gupta AK, Deorari AK, Paul VK, Sreenivas V. Trial of daily vitamin D supplementation in preterm infants. *Pediatrics.* Vol 1332014:3.

**126.** Nader NS, Aguirre Castaneda R, Wallace J, Singh R, Weaver A, Kumar S. Effect of vitamin D3 supplementation on serum 25(OH)D, lipids and markers of insulin resistance in obese adolescents: a prospective, randomized, placebo-controlled pilot trial. *Horm Res Paediatr.* Vol 822014:107-112.

**127.** Mondal K, Seth A, Marwaha RK, Dhanwal D, Aneja S, Singh R, Sonkar P. A Randomized controlled trial on safety and efficacy of single intramuscular versus staggered oral dose of 600 000IU Vitamin D in treatment of nutritional rickets. *J Trop Pediatr.* Vol 602014:203-210.

**128.** Mittal H, Rai S, Shah D, Madhu SV, Mehrotra G, Malhotra RK, Gupta P. 300,000 IU or 600,000 IU of oral vitamin D3 for treatment of nutritional rickets: a randomized controlled trial. *Indian Pediatr.* Vol 512014:265-272.

**129.** Marwaha RK, Tandon N, Agarwal N, Puri S, Agarwal R, Singh S, Mani K. Impact of two regimens of vitamin D supplementation on calcium - vitamin D - PTH axis of schoolgirls of Delhi. *Indian Pediatr.* Vol 472010:761-769.

**130.** Marchisio P, Consonni D, Baggi E, Zampiero A, Bianchini S, Terranova L, Tirelli S, Esposito S, Principi N. Vitamin D supplementation reduces the risk of acute otitis media in otitis-prone children. *Pediatr.Infect.Dis.J.* Vol 322013:1055-1060.

**131.** Manaseki-Holland S, Qader G, Isaq Masher M, Bruce J, Zulf Mughal M, Chandramohan D, Walraven G. Effects of vitamin D supplementation to children diagnosed with pneumonia in Kabul: a randomised controlled trial. *Trop. Med. Int. Health.* Vol 152010:1148-1155.

**132.** Manaseki-Holland S, Maroof Z, Bruce J, Mughal MZ, Masher MI, Bhutta ZA, Walraven G, Chandramohan D. Effect on the incidence of pneumonia of vitamin D supplementation by quarterly bolus dose to infants in Kabul: a randomised controlled superiority trial. *Lancet.* Vol 3792012:1419-1427.

**133.** Mallet E, Philippe F, Castanet M, Basuyau J-P. [Administration of a single Winter oral dose of 200,000 IU of vitamin D3 in adolescents in Normandy: evaluation of the safety and vitamin D status obtained]. *Arch Pediatr.* Vol 172010:1042-1046.

**134.** Lewis RD, Laing EM, Hill Gallant KM, Hall DB, McCabe GP, Hausman DB, Martin BR, Warden SJ, Peacock M, Weaver CM. A randomized trial of vitamin D₃ supplementation in children: dose-response effects on vitamin D metabolites and calcium absorption. *J Clin Endocrinol Metab.* Vol 982013:4816-4825.

**135.** Lewis E, Fernandez C, Nella A, Hopp R, Gallagher JC, Casale TB. Relationship of 25-hydroxyvitamin D and asthma control in children. *Ann. Allergy Asthma Immunol.* Vol 1082012:281-282.

**136.** Kumar GT, Sachdev HS, Chellani H, Rehman AM, Singh V, Arora H, Filteau S. Effect of weekly vitamin D supplements on mortality, morbidity, and growth of low birthweight term infants in India up to age 6 months: randomised controlled trial. *BMJ.* Vol 3422011:d2975.

**137.** Khadgawat R, Marwaha RK, Garg MK, Ramot R, Oberoi AK, Sreenivas V, Gahlot M, Mehan N, Mathur P, Gupta N. Impact of vitamin D fortified milk supplementation on vitamin D status of healthy school children aged 10-14 years. *Osteoporos.Int.* Vol 242013:2335-2343.

**138.** Kelishadi R, Salek S, Salek M, Hashemipour M, Movahedian M. Effects of vitamin D supplementation on insulin resistance and cardiometabolic risk factors in children with metabolic syndrome: a triple-masked controlled trial. *J Pediatr (Rio J).* Vol 902014:28-34.

**139.** Kari JA, Baghdadi OT, El-Desoky S. Is high-dose cholecalciferol justified in children with chronic kidney disease who failed low-dose maintenance therapy? *Pediatr Nephrol.* Vol 282013:933-937.

**140.** Kakalia S, Sochett EB, Stephens D, Assor E, Read SE, Bitnun A. Vitamin D supplementation and CD4 count in children infected with human immunodeficiency virus. *J Pediatr.* Vol 1592011:951-957.

**141.** Javed A, Vella A, Balagopal PB, Fischer PR, Weaver AL, Piccinini F, Dalla Man C, Cobelli C, Giesler PD, Laugen JM, Kumar S. Cholecalciferol Supplementation Does Not Influence β-Cell Function and Insulin Action in Obese Adolescents: A Prospective Double-Blind Randomized Trial. *J Nutr.* Vol 1452015:284-290.

**142.** Holmlund-Suila E, Viljakainen H, Hytinantti T, Lamberg-Allardt C, Andersson S, Mäkitie O. High-dose vitamin d intervention in infants--effects on vitamin d status, calcium homeostasis, and bone strength. *J Clin Endocrinol Metab.* Vol 972012:4139-4147.

**143.** Hill KM, Laing EM, Hausman DB, Acton A, Martin BR, McCabe GP, Weaver CM, Lewis RD, Peacock M. Bone turnover is not influenced by serum 25-hydroxyvitamin D in pubertal healthy black and white children. *Bone.* Vol 512012:795-799.

**144.** Hari P, Gupta N, Hari S, Gulati A, Mahajan P, Bagga A. Vitamin D insufficiency and effect of cholecalciferol in children with chronic kidney disease. *Pediatr Nephrol.* Vol 252010:2483-2488.

**145.** Grant CC, Stewart AW, Scragg R, Milne T, Rowden J, Ekeroma A, Wall C, Mitchell EA, Crengle S, Trenholme A, Crane J, Camargo CA. Vitamin D during pregnancy and infancy and infant serum 25-hydroxyvitamin D concentration. *Pediatrics.* Vol 1332014:e143-153.

**146.** Ghazi AA, Hosseinpanah F, M Ardakani E, Ghazi S, Hedayati M, Azizi F. Effects of different doses of oral cholecalciferol on serum 25(OH)D, PTH, calcium and bone markers during fall and winter in schoolchildren. *Eur J Clin Nutr.* Vol 642010:1415-1422.

**147.** Garg MK, Marwaha RK, Khadgawat R, Ramot R, Obroi AK, Mehan N, Gupta N, Madan R. Efficacy of vitamin D loading doses on serum 25-hydroxy vitamin D levels in school going adolescents: an open label non-randomized prospective trial. *J. Pediatr. Endocrinol. Metab.* Vol 262013:515-523.

**148.** Ganmaa D, Giovannucci E, Bloom BR, Fawzi W, Burr W, Batbaatar D, Sumberzul N, Holick MF, Willett WC. Vitamin D, tuberculin skin test conversion, and latent tuberculosis in Mongolian school-age children: a randomized, double-blind, placebo-controlled feasibility trial. *Am J Clin Nutr.* Vol 962012:391-396.

**149.** Gallo S, Comeau K, Vanstone C, Agellon S, Sharma A, Jones G, L’Abbé M, Khamessan A, Rodd C, Weiler H. Effect of different dosages of oral vitamin D supplementation on vitamin D status in healthy, breastfed infants: a randomized trial. *JAMA.* Vol 3092013:1785-1792.

**150.** Emel T, Doğan DA, Erdem G, Faruk O. Therapy strategies in vitamin D deficiency with or without rickets: efficiency of low-dose stoss therapy. *J. Pediatr. Endocrinol. Metab.* Vol 252012:107-110.

**151.** Ekbote VH, Khadilkar AV, Chiplonkar SA, Hanumante NM, Khadilkar VV, Mughal MZ. A pilot randomized controlled trial of oral calcium and vitamin D supplementation using fortified laddoos in underprivileged Indian toddlers. *Eur J Clin Nutr.* Vol 652011:440-446.

**152.** Ekbote V, Khadilkar A, Chiplonkar S, Mughal Z, Khadilkar V. Enhanced effect of zinc and calcium supplementation on bone status in growth hormone-deficient children treated with growth hormone: a pilot randomized controlled trial. *Endocrine.* Vol 432013:686-695.

**153.** Dong Y, Stallmann-Jorgensen IS, Pollock NK, Harris RA, Keeton D, Huang Y, Li K, Bassali R, Guo D-h, Thomas J, Pierce GL, White J, Holick MF, Zhu H. A 16-week randomized clinical trial of 2000 international units daily vitamin D3 supplementation in black youth: 25-hydroxyvitamin D, adiposity, and arterial stiffness. *J Clin Endocrinol Metab.* Vol 952010:4584-4591.

**154.** Doğan M, Cesur Y, Zehra Doğan Ş, Kaba S, Bulan K, Cemek M. Oxidant/antioxidant system markers and trace element levels in children with nutritional rickets. *J. Pediatr. Endocrinol. Metab.* Vol 252012:1129-1139.

**155.** Choudhary N, Gupta P. Vitamin D supplementation for severe pneumonia--a randomized controlled trial. *Indian Pediatr.* Vol 492012:449-454.

**156.** Cayir A, Turan MI, Ozkan O, Cayir Y, Kaya A, Davutoglu S, Özkan B. Serum vitamin D levels in children with recurrent otitis media. *Eur Arch Otorhinolaryngol.* Vol 2712014:689-693.

**157.** Carnes J, Quinn S, Nelson M, Jones G, Winzenberg T. Intermittent high-dose vitamin D corrects vitamin D deficiency in adolescents: a pilot study. *Eur J Clin Nutr.* Vol 662012:530-532.

**158.** Camargo Jr CA, Ganmaa D, Sidbury R, Erdenedelger K, Radnaakhand N, Khandsuren B. Randomized trial of vitamin D supplementation for winter-related atopic dermatitis in children. *J Allergy Clin Immunol.* Vol 1342014:831-835.

**159.** Belenchia AM, Tosh AK, Hillman LS, Peterson CA. Correcting vitamin D insufficiency improves insulin sensitivity in obese adolescents: a randomized controlled trial. *Am J Clin Nutr.* Vol 972013:774-781.

**160.** Baris S, Kiykim A, Ozen A, Tulunay A, Karakoc-Aydiner E, Barlan IB. Vitamin D as an adjunct to subcutaneous allergen immunotherapy in asthmatic children sensitized to house dust mite. *Allergy.* Vol 692014:246-253.

**161.** Ashraf AP, Alvarez JA, Gower BA, Saenz KH, McCormick KL. Associations of serum 25-hydroxyvitamin D and components of the metabolic syndrome in obese adolescent females. *Obesity (Silver Spring).* Vol 192011:2214-2221.

**162.** Arpadi SM, McMahon DJ, Abrams EJ, Bamji M, Purswani M, Engelson ES, Horlick M, Shane E. Effect of supplementation with cholecalciferol and calcium on 2-y bone mass accrual in HIV-infected children and adolescents: a randomized clinical trial. *Am J Clin Nutr.* Vol 952012:678-685.

**163.** Aluisio AR, Maroof Z, Chandramohan D, Bruce J, Mughal MZ, Bhutta Z, Walraven G, Masher MI, Ensink JHJ, Manaseki-Holland S. Vitamin D₃supplementation and childhood diarrhea: a randomized controlled trial. *Pediatrics.* Vol 1322013:e832-840.

**164.** Alonso A, Rodríguez J, Carvajal I, Prieto MAL, Rodríguez RMA, Pérez AMA, Cepeda A, Nuño F, Santos F, Asturias CGoPwVDi. Prophylactic vitamin D in healthy infants: assessing the need. *Metab. Clin. Exp.* Vol 602011:1719-1725.

**165.** Alizadeh TP, Sajjadian N, Beyrami B, Shariat M. Prophylactic effect of low dose vitamin D in osteopenia of prematurity: a clinical trial study. *Acta Med Iran.* Vol 522014:671-674.

**166.** Al-Shaar L, Mneimneh R, Nabulsi, Maalouf J, Fuleihan GE-H. Vitamin D3 dose requirement to raise 25-hydroxyvitamin D to desirable levels in adolescents: results from a randomized controlled trial. *J Bone Miner.Res.* Vol 292014:944-951.

**167.** Aguirre Castaneda R, Nader N, Weaver A, Singh R, Kumar S. Response to vitamin D3 supplementation in obese and non-obese Caucasian adolescents. *Horm Res Paediatr.* Vol 782012:226-231.

**168.** Aggarwal V, Seth A, Marwaha RK, Sharma B, Sonkar P, Singh S, Aneja S. Management of nutritional rickets in Indian children: a randomized controlled trial. *J Trop Pediatr.* Vol 592013:127-133.

**169.** Abrams SA, Hawthorne KM, Chen Z. Supplementation with 1000 IU vitamin D/d leads to parathyroid hormone suppression, but not increased fractional calcium absorption, in 4-8-y-old children: a double-blind randomized controlled trial. *Am J Clin Nutr.* Vol 972013:217-223.
